# Nomogram combining dual-energy computed tomography features and radiomics for differentiating parotid warthin tumor from pleomorphic adenoma: a retrospective study

**DOI:** 10.3389/fonc.2025.1505385

**Published:** 2025-03-04

**Authors:** Zhiwei Gong, Jianying Li, Yilin Han, Shiyu Chen, Lijun Wang

**Affiliations:** ^1^ Department of Radiology, The First Affiliated Hospital of Dalian Medical University, Dalian, China; ^2^ CT Imaging Research Center, GE Healthcare, Shanghai, China

**Keywords:** parotid tumor, dual-energy computed tomography, radiomics, machine learning, combined nomogram, identification

## Abstract

**Introduction:**

Accurate differentiation between pleomorphic adenomas (PA) and Warthin tumors (WT) in the parotid gland is challenging owing to overlapping imaging features. This study aimed to evaluate a nomogram combining dual-energy computed tomography (DECT) quantitative parameters and radiomics to enhance diagnostic precision.

**Methods:**

This retrospective study included 120 patients with pathologically confirmed PA or WT, randomly divided into training and test sets (7:3). DECT features, including tumor CT values from 70 keV virtual monochromatic images (VMIs), iodine concentration (IC), and normalized IC (NIC), were analyzed. Independent predictors were identified via logistic regression. Radiomic features were extracted from segmented regions of interest and filtered using the K-best and least absolute shrinkage and selection operator. Radiomic models based on 70 keV VMIs and material decomposition images were developed using logistic regression (LR), support vector machine (SVM), and random forest (RF). The best-performing radiomics model was combined with independent DECT predictors to construct a model and nomogram. Model performance was assessed using ROC curves, calibration curves, and decision curve analysis (DCA).

**Results:**

IC (venous phase), NIC (arterial phase), and NIC (venous phase) were independent DECT predictors. The DECT feature model achieved AUCs of 0.842 and 0.853 in the training and test sets, respectively, outperforming the traditional radiomics model (AUCs 0.836 and 0.834, respectively). The DECT radiomics model using arterial phase water-based images with LR showed improved performance (AUCs 0.883 and 0.925). The combined model demonstrated the highest discrimination power, with AUCs of 0.910 and 0.947. The combined model outperformed the DECT features and conventional radiomics models, with AUCs of 0.910 and 0.947, respectively (P<0.05). While the difference in AUC between the combined model and the DECT radiomics model was not statistically significant (P>0.05), it showed higher specificity, accuracy, and precision. DCA found that the nomogram gave the greatest net therapeutic effect across a broad range of threshold probabilities.

**Discussion:**

The nomogram combining DECT features and radiomics offers a promising non-invasive tool for differentiating PA and WT in clinical practice.

## 1 Introduction

Parotid gland tumors represent approximately 2–3% of all head and neck tumors, with approximately 80% being benign (1). Among them, pleomorphic adenoma (PA) is the most common, constituting a significant portion of parotid gland tumors of epithelial origin. Although typically benign, approximately 3.2% of PAs have the potential for malignant transformation (2, 3). In addition, PA can infiltrate surrounding tissues, forming pseudopods or satellite nodules, making complete excision using a traditional debulking surgery challenging. This results in a risk of recurrence, necessitating treatment via extracapsular dissection (ECD) (4, 5). Warthin tumors (WT) account for approximately 15% of primary parotid tumors, making them the second most common after PA (6). WT predominantly affects middle-aged men and smokers, with some cases involving both parotid glands. Although malignant transformation and recurrence are very rare (7), WT often triggers inflammatory reactions in surrounding tissues, complicating surgical intervention. Consequently, conservative treatment is typically preferred to avoid unnecessary surgical risks (8, 9). Therefore, differentiating between PA and WT is crucial because it directly influences clinical decision-making and individualized surgical planning.

Fine-needle aspiration Biopsy (FNAB) is the current “gold standard” for tumor diagnosis. However, it is an invasive procedure with risks, including potential damage to the facial nerve, which can lead to peripheral facial paralysis (10, 11). Ultrasound is effective in detecting tumors in the superficial lobes of the parotid gland, but visualizing deeper lesions can be challenging, and its accuracy is highly dependent on the operator’s experience (12). While magnetic resonance imaging (MRI) offers high tissue resolution, its use is limited by factors such as prolonged examination times and sensitivity to metal implants, making it less ideal as a primary diagnostic tool (13–16). Traditional computed tomography scans provide detailed information about the tumor’s anatomical location and lesion extent and can uniquely assess calcification and adjacent vascular conditions (17, 18). However, traditional CT relies on attenuation-based imaging, which lacks material-specific resolution.

Dual-energy CT (DECT) addresses this limitation by using dual-energy spectra acquired simultaneously during CT scanning to improve the analysis of physical and chemical properties. One common method for obtaining dual-energy spectra is the instantaneous switching between high and low tube voltages (140 kVp and 80 kVp) during data acquisition. DECT works by converting the absorption data of the same material at different energy levels into density data based on the variation in attenuation coefficients of different substances exposed to varying X-ray energy levels. This process produces material decomposition (MD) images (19). Given that iodine is the primary material used in contrast agents for enhanced scanning, measuring iodine concentration on water-iodide MD images allows for a detailed visualization of iodine distribution, which can reflect blood perfusion at lesion sites (20).

Radiomics involves using machine learning techniques to extract information from medical images that are not visible to the naked eye, capturing the microscopic characteristics of lesions. This allows for predicting the biological behavior of lesions, as well as patient prognosis and treatment outcomes. As a novel biomarker, radiomics is valuable in differentiating tumor types by assessing their heterogeneity (21). Radiomics has shown great promise in improving noninvasive individualized treatment and monitoring by differentiating parotid tumor types, evaluating the integrity of benign tumor envelopes, grading malignant tumors, and differentiating lymphoid lesions, and these studies use a variety of imaging techniques such as CT, MRI, US, and PET-CT (22–26). However, the current issue is the small number and varying quality of studies in this direction, with none of them split at several time points, prospectively verified, or cost-effective. The majority of the studies did not disclose public data, and one of the issues was the difficulty in reproducing the study (27). As a result, the clinical application needs to be further investigated. In this work, we included the energy spectrum CT substance separation image radiomics features and compared them to the performance of traditional CT histology, with the goal of providing evidence for the validity of radiomics in the parotid gland using a novel method.

The integration of radiomics with DECT has been increasingly applied to disease identification and prediction across various organ systems (28–30). However, its application to the parotid gland remains limited. In one study, Ajmi et al. used virtual monochromatic images from DECT combined with radiomics analysis to differentiate between PA and WT. They found that combining radiomics features from monochromatic images at multiple energy levels significantly improved diagnostic accuracy compared to using a radiomics model based on a single energy level (65 keV) (31). However, the study did not explore the potential value of radiomics features derived from MD images. We hypothesized that radiomics based on MD images from DECT, which quantitatively analyze iodine concentration differences between lesions, along with microscopic image analysis, could provide a novel approach for identifying parotid tumors in clinical practice. Therefore, this study aimed to assess the value of DECT MD image-based radiomics and evaluate the effectiveness of a nomogram that combines radiomics with DECT-independent predictors in differentiating the two most common parotid tumors, playing an adjunctive role in the development of patient treatment strategies using a non-invasive modality.

## 2 Materials and methods

### 2.1 Patient population

**Figure 1:**
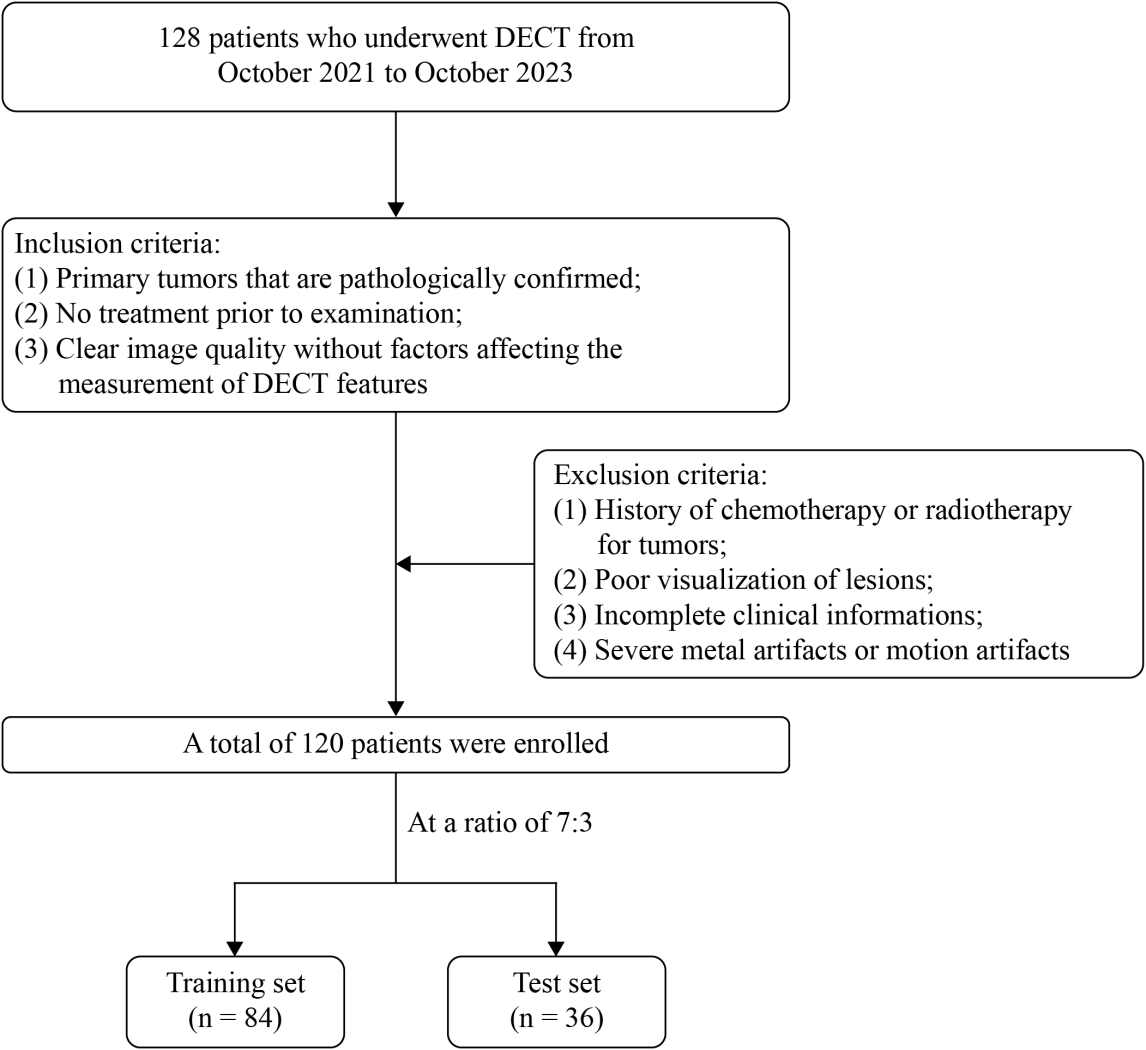
Flowchart of patient selection.

This retrospective study, which did not interfere with the clinical diagnosis or treatment of any patients, was approved by the Ethics Committee of the First Affiliated Hospital of Dalian Medical University (approval number: PJ-KS-KY-2024-230; date: April 3, 2024), with a waiver for written informed consent. We collected data from 128 patients diagnosed with PA and WT of the parotid gland who presented to our institution and underwent DECT enhancement between October 2021 and October 2023. The inclusion criteria were as follows: (1) primary parotid tumors with all types confirmed by surgical pathology; (2) no prior treatment before the CT examination; and (3) high-quality CT images without factors affecting the measurement of DECT parameters. The exclusion criteria were as follows: (1) history of chemotherapy or radiotherapy for parotid tumors; (2) poorly visible lesions on imaging; (3) incomplete clinical data; and (4) severe metallic or motion artifacts in images. After applying these criteria, we excluded one patient with extensive metal (denture) artifacts obscuring the tumor, two with poorly visualized lesions, and five with missing clinical data. This resulted in a total of 120 patients being included in the study, comprising 66 cases of PA and 54 cases of WT. The patients were randomly assigned to training and test sets in a 7:3 ratio, with 84 cases in the training set and 36 cases in the test set. The patient selection process is illustrated in **Figure 1**.

### 2.2 DECT image acquisition

All patients underwent non-contrast and contrast-enhanced scans from the superior orbital rim to the level of the aortic arch using a GE Discovery CT 750 HD scanner. The scanning parameters were as follows: instantaneous switching between high (140 kVp) and low (80 kVp) tube voltages, a tube current of 600 mA, a gantry rotation speed of 0.6 seconds per rotation, a collimator width of 64 × 0.625 mm, and a slice thickness and spacing of 2.5 mm. The contrast agent used was iohexol (300 mg I/mL) administered at a dose of 100–200 mL via an elbow vein injection. DECT scans in the arterial phase (AP) and venous phase (VP) were initiated at 30 s and 60 s, respectively, after contrast injection. Iodine- and water-based MD sequences of the non-contrast (NP), arterial, and venous phases were reconstructed using Gemstone Spectral Imaging (GSI) post-processing software. The images were then imported into the picture archiving and communication system (PACS) for analysis.

### 2.3 DECT characterization and assessment

**Figure 2:**
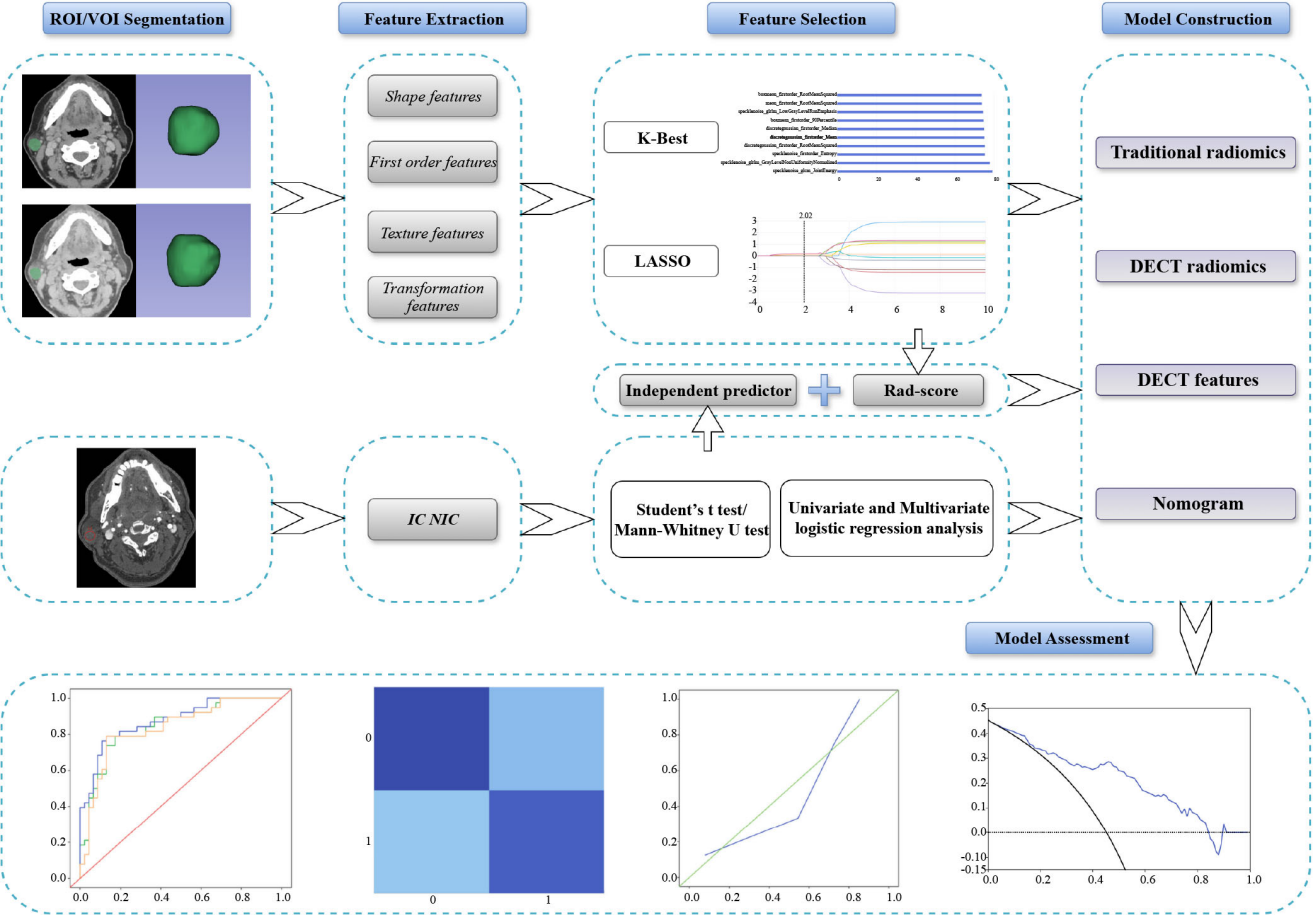
Overview of the study workflow of this study. DECT, dual-energy computed tomography; IC, iodine concentration; LASSO, least absolute shrinkage and selection operator; NIC, normalized iodine concentration; ROI, region of interest; VOI, volume of interest.

**Figure 3:**
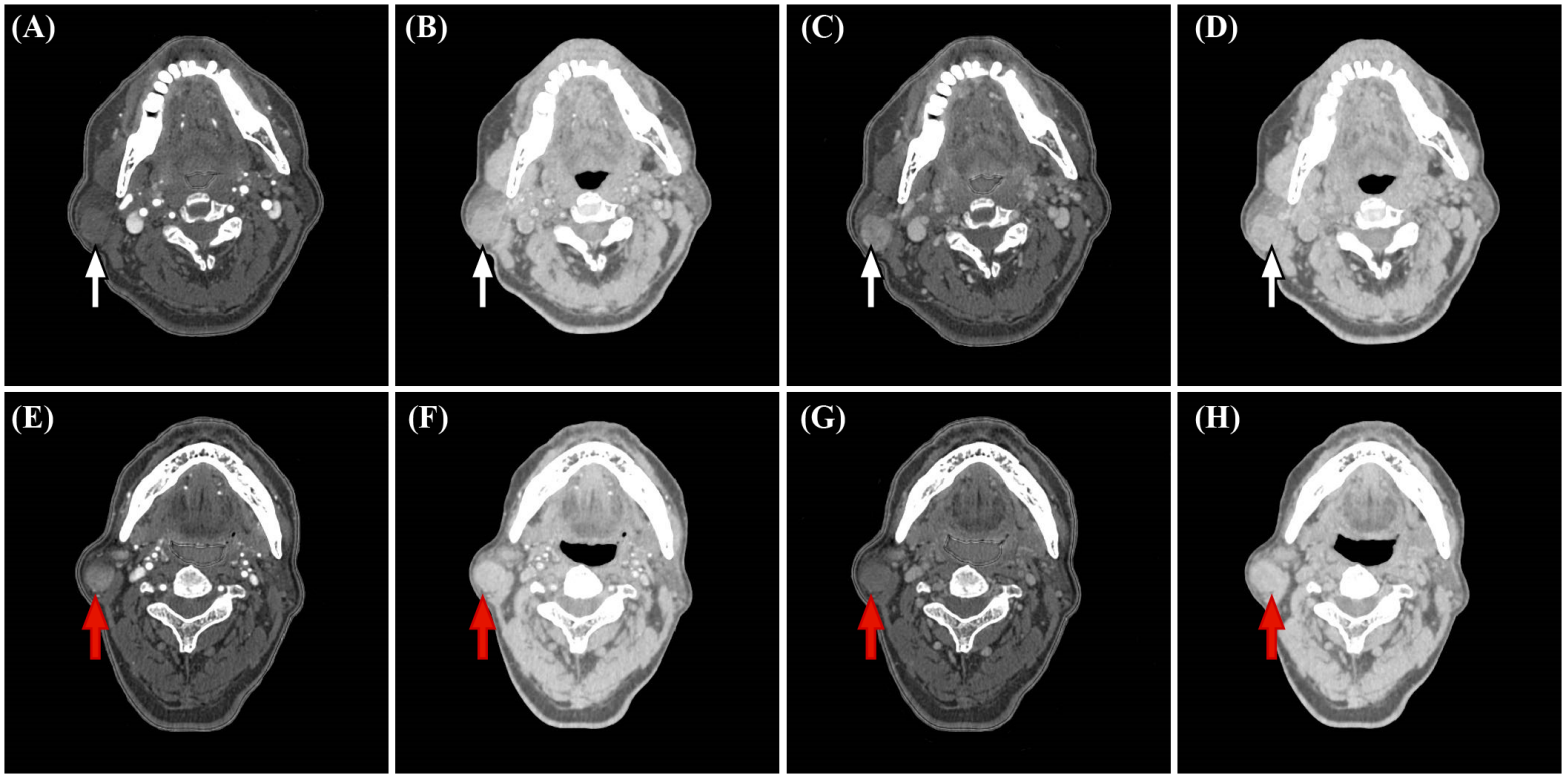
Representative dual-energy CT (DECT) material decomposition (MD) images. (white arrows, **A-D**) PA in a 67-year-old woman. (red arrows, **E-H**) WT in a 71-year-old man. **(A, E)** Iodine-based MD images in the arterial phase. **(B, F)** Water-based MD images in the arterial phase. **(C, G)** Iodine-based MD images in the venous phase. **(D, H)**. Water-based MD images in the venous phase.


The study workflow is outlined in **Figure 2**. Image processing and data measurements were performed using GSI Viewer 4.5 software (GE Healthcare). Key features such as the number, location, maximum diameter of the lesions, cystic changes, calcifications, and lymph node status were recorded and analyzed (**Table 1**). As illustrated in **Figure 3**, we examined 70 keV virtual monochromatic images (VMIs) and MD images (iodine-based and water-based). A radiologist with three years of experience in head and neck imaging, blinded to all pathological findings, measured the DECT quantitative parameters by placing regions of interest (ROIs) within the tumor and the ipsilateral common carotid artery. The ROIs were positioned to encompass the entire tumor while avoiding calcifications and prominent enhanced vessels. Measurements were centered at the level of maximum lesion display, averaged across three consecutive slices. Lesion attenuation (in Hounsfield units, HU) was obtained from the 70 keV VMIs for the NP, AP, and VP. Iodine concentrations for the tumor and common carotid artery were assessed using iodine-based MD images. The degree of enhancement and normalized iodine concentration (NIC) were calculated using the following formulas:

**Table 1:** Clinical data of patients with PA and WT.

Clinical characteristics	PA n = 66	WT n = 54	p value
Age, years ^a^	47.23 ± 13.57	61.22 ± 8.20	< 0.001
Sex (male/female) ^b^	28/38	50/4	< 0.001
Smoking history (present/absent) ^b^	22/44	46/8	< 0.001
Number of lesions (single/multiple) ^b^	65/1	42/12	0.001
Location (right/left) ^b^	39/27	30/24	0.697
Deep lobe involvement (present/absent) ^b^	8/58	2/52	0.182
Cystic lesions (present/absent) ^b^	18/48	21/33	< 0.001
Calcification (present/absent) ^b^	2/64	1/53	0.999
Maximum diameter of the tumor, cm ^c^	2.00 (1.40, 2.70)	2.40 (2.00, 2.90)	0.008
Minor diameter of lymph node, cm ^c^	0.60 (0.50, 0.70)	0.60 (0.60, 0.80)	0.004

PA, pleomorphic adenoma; WT, Warthin tumors; a, Student’s t test; b, χ^2^ or Fisher’s exact test; c, Mann–Whitney U test.

$$\begin{array}{c} {\text{IC}}_{\text{CCA}}Enhancement\;degree\\ =attenuation\;in\;AP\;or\;VP–attenuation\;in\;NP \end{array}$$

$$\begin{array}{l} NIC=iodine\;concentration\;in\;the\;lesion\left(IC\_lesion\right)\\ /iodine\;concentration\;in\;the\;ipsilateral\;common\;carotid\;artery\;\\ \left(IC\_CCA\right) \end{array}$$

The largest lesion was selected for analysis in cases with multiple parotid lesions. Differences between WT and PA were analyzed using univariate logistic regression. Significant variables (p<0.05) were included in a multivariate logistic regression model to identify independent predictors of DECT features and calculate odds ratios (ORs) with 95% confidence intervals (CIs).

### 2.4 Segmentation of tumor ROI

Lesions were manually segmented using the open-source software 3D Slicer (https://www.slicer.org). ROIs were delineated slice by slice to generate the volume of interest (VOI), carefully avoiding adjacent tissues and blood vessels. Layers heavily affected by artifacts, such as those near the mandible, were excluded from the segmentation. To assess intra- and inter-observer consistency, 30 patients (18 PA, 12 WT) were randomly selected, and their VOIs were re-segmented after one month by the same radiologist to evaluate intra-observer agreement. Another radiologist independently repeated the segmentation to assess inter-observer agreement. The reproducibility of radiomic features was quantified using the intraclass correlation coefficient (ICC).

### 2.5 Radiomics feature extraction and filtering

The segmented VOIs were imported into the United Imaging Intelligence workstation (Shanghai, China). All sequences were aligned and normalized in terms of window width and level. A total of 2,048 features were extracted from each VOI, categorized into (1) Shape-based (2D/3D) features; (2) First-order histogram features; and (3) Second-order texture features, including gray level run length matrix (GLRLM), gray level co-occurrence matrix (GLCM), gray level dependence matrix (GLDM), gray level size zone matrix (GLSZM), and neighboring gray-tone difference matrix (NGTDM). These first- and second-order features were extracted not only from the original image but also from derived images processed with 14 filters, such as Mean, Bilateral, and Speckle Noise. The algorithms used for feature extraction follow the Image Biomarker Standardization Initiative (IBSI) guidelines (32).

Features with intra- and inter-observer ICC values greater than 0.75 were retained and standardized using z-score normalization. The optimal regularization parameters were determined through five-fold cross-validation in the training set. Univariate analysis was conducted using the K-best method with F-value tests to identify significant differences between classifications, selecting the top 10 most important features. These features were further refined using the least absolute shrinkage and selection operator (LASSO) analysis based on the feature coefficients at the maximum alpha value. The final model was trained using the selected features, and its performance and generalization ability were evaluated with the test set.

### 2.6 Development and assessment of models

Predictive models were developed using logistic regression (LR), support vector machine (SVM), and random forest (RF) algorithms based on independent DECT predictors and filtered radiomic features. The diagnostic performance of each model was assessed by comparing the area under the curve (AUC) of receiver operating characteristic (ROC), as well as sensitivity, specificity, accuracy, and precision. Significant DECT features were utilized to construct the DECT-based model. Among the radiomics model, the best-performing model from the 70 keV non-contrast and contrast-enhanced sequences was selected as the conventional radiomics model. In contrast, the top-performing model based on MD images was selected as the DECT radiomics model. A combined model was developed by calculating the Rad-score from the features of the best MD model and integrating it with independent DECT predictors. A nomogram was constructed for this combined model. Calibration curves were generated to determine the goodness-of-fit between predicted and actual values across the four models. Decision curve analysis (DCA) was performed to assess the net benefit rates of each model, providing insights into their clinical utility.

### 2.7 Statistical analysis

Statistical analyses were conducted using SPSS software (version 26.0, IBM) and MedCalc software (version 22.009, https://www.medcalc.org). Quantitative parameters following a normal distribution are presented as mean ± standard deviation (x ± s) and were compared using the Student’s t-test. Non-normally distributed data are expressed as median and interquartile range [M (P25, P75)] and were analyzed using the Mann–Whitney U test to assess between-group differences. Qualitative data were analyzed using the chi-square test or Fisher’s exact test, depending on the data characteristics. Univariate and multivariate logistic regression analyses were employed to evaluate all parameters, with significant variables sequentially filtered to identify independent predictors. The Delong test was used to compare the differences in AUC values between the models, with a p-value of less than 0.05 considered statistically significant.

## 3 Results

### 3.1 Patient information

A total of 120 patients (78 males, 42 females) were included in this study, ranging in age from 24 to 78 years. Among them, 66 had PA and 54 WT. Detailed clinical data for both groups are presented in **Table 1**. Statistically significant differences were observed between PA and WT patients in terms of age, sex, smoking history, number of tumors, presence of cystic lesions, maximum lesion diameter, and ipsilateral lymph node status (all p<0.05).

### 3.2 DECT parameter analysis and feature model development

As presented in **Supplementary Table S1**, no significant differences in DECT features were observed between the training and test sets. Within the training set, significant differences were observed between PA and WT in attenuation (NP and AP), enhancement degree (AP and VP), IC, and NIC (all p<0.05). Specifically, NP attenuation, AP attenuation, AP enhancement degree, IC, and NIC were significantly higher in WT than in PA. However, the VP enhancement degree, IC, and NIC were notably lower in WT (**Table 2**).

**Table 2:** DECT features of PA and WT.

DECT features	Training set (n = 84)			Test set (n = 36)		
	PA (n = 46)	WT (n = 38)	p value	PA (n = 20)	WT (n = 16)	p value
Attenuation (NP), HU ^b^	32.44 ± 9.81	45.07 ± 9.54	<0.001	33.73 ± 13.39	49.14 ± 9.85	0.003
Attenuation (AP), HU ^b^	53.44 ± 23.86	81.74 ± 23.46	<0.001	46.88 ± 20.81	84.04 ± 36.12	< 0.001
Attenuation (VP), HU ^b^	71.92 ± 26.12	75.72 ± 11,51	0.200	70.35 ± 21.92	86.43 ± 14.59	0.017
Enhancement degree (AP), HU ^a^	14.79 (6.03, 27.78)	32.27 (17.36, 55.00)	0.001	8.64 (4.47, 18.34)	31.44 (9.25, 46.42)	0.016
Enhancement degree (VP), HU ^a^	38.72 (29.39, 48.70)	29.95 (22.75, 39.32)	0.012	30.70 (28.92, 44.33)	34.72 (29.72, 48.58)	0.464
IC (NP), 100 μg/cm³ ^b^	-9.42 ± 2.87	-10.14 ± 2.31	0.036	-10.11 ± 2.33	-8.70 ± 2.14	0.068
IC (AP), 100 μg/cm³ ^a^	-3.28 (-6.67, 2.46)	1.37 (-2.38, 7.62)	0.003	-4.23 (-8.32, -1.78)	2.67 (-4.71, 11.54)	0.005
IC (VP), 100 μg/cm³ ^a^	3.17 (-1.69, 10.96)	-0.48 (-3.87, 2.64)	0.016	-0.72 (-2.72, 5.17)	0.98 (-3.01, 4.39)	0.987
NIC (AP), % ^a^	-3.95 (-7.10, 2.79)	1.76 (-3.34, 8.89)	0.016	-5.41 (-9.60, -1.54)	3.19 (-5.12, 17.66)	0.013
NIC (VP), % ^b^	14.98 ± 34.02	-5.42 ± 26.38	0.003	1.51 ± 30.66	1.25 ± 18.19	0.977

PA, pleomorphic adenoma; WT, Warthin tumors; DECT, dual-energy CT; HU, Hounsfield unit; NP, non-enhanced phase; AP, arterial phase; VP, venous phase; IC, iodine concentration; NIC, normalized iodine concentration; a, Mann–Whitney U test; b, Student’s t-test.

Logistic regression analysis (**Table 3**) identified IC (VP), NIC (AP), and NIC (VP) as independent predictors for distinguishing PA from WT. When these features were combined to construct the model, no significant performance differences were observed among the LR, SVM, and RF models (**Supplementary Table S2** and **Supplementary Figure S1**). Given its relatively better performance in the test set, the LR model was ultimately selected as the DECT feature model.

**Table 3:** Univariate and multivariate logistic regression analyses of DECT features.

DECT features	Univariate logistic regression analysis			Multivariate logistic regression analysis		
	p value	OR	95% CI	p value	OR	95% CI
Attenuation (NP), HU ^a^	< 0.001	1.135	1.081–1.192	0.749	NA	
Attenuation (AP), HU ^a^	< 0.001	1.050	1.030–1.070	0.747		
Attenuation (VP), HU ^a^	0.054	NA				
Enhancement degree (AP), HU ^a^	< 0.001	1.039	1.019–1.060	0.748		
Enhancement degree (VP), HU ^a^	0.072	NA				
IC (NP), 100 μg/cm³ ^b^	0.866	NA				
IC (AP), 100 μg/cm³ ^a^	< 0.001	1.104	1.046–1.165	0.053		
IC (VP), 100 μg/cm³ ^a^	0.005	0.912	0.855–0.973	0.001	0.362	0.198–0.663
NIC (AP), % ^a^	0.002	1.065	1.023–1.108	0.006	0.642	0.470–0.878
NIC (VP), % ^a^	0.013	0.983	0.970–0.996	0.022	1.112	1.016–1.218

DECT, dual-energy CT; OR, odds ratio; CI, confidence interval; NA, not available; NP, non-enhanced phase; AP, arterial phase; VP, venous phase; IC, iodine concentration; NIC, normalized iodine concentration; a, Mann–Whitney U test; b, Student’s t-test.

### 3.3 Traditional radiomics feature extraction and model development

From the 70 keV VMIs across the non-contrast, arterial, and venous phases, 784, 398, and 536 highly reproducible features (ICC>0.75) were extracted, respectively. Subsequent K-best and LASSO analyses retained two, four, and four non-zero coefficient features for each phase, respectively. **Supplementary Table S3** provides details on the selected features and their corresponding LASSO coefficients. Diagnostic models were constructed using LR, SVM, and RF algorithms based on these selected features, with model performance detailed in **Supplementary Table S4** and illustrated in **Supplementary Figures S2**-**S4**.

**Figure 4:**
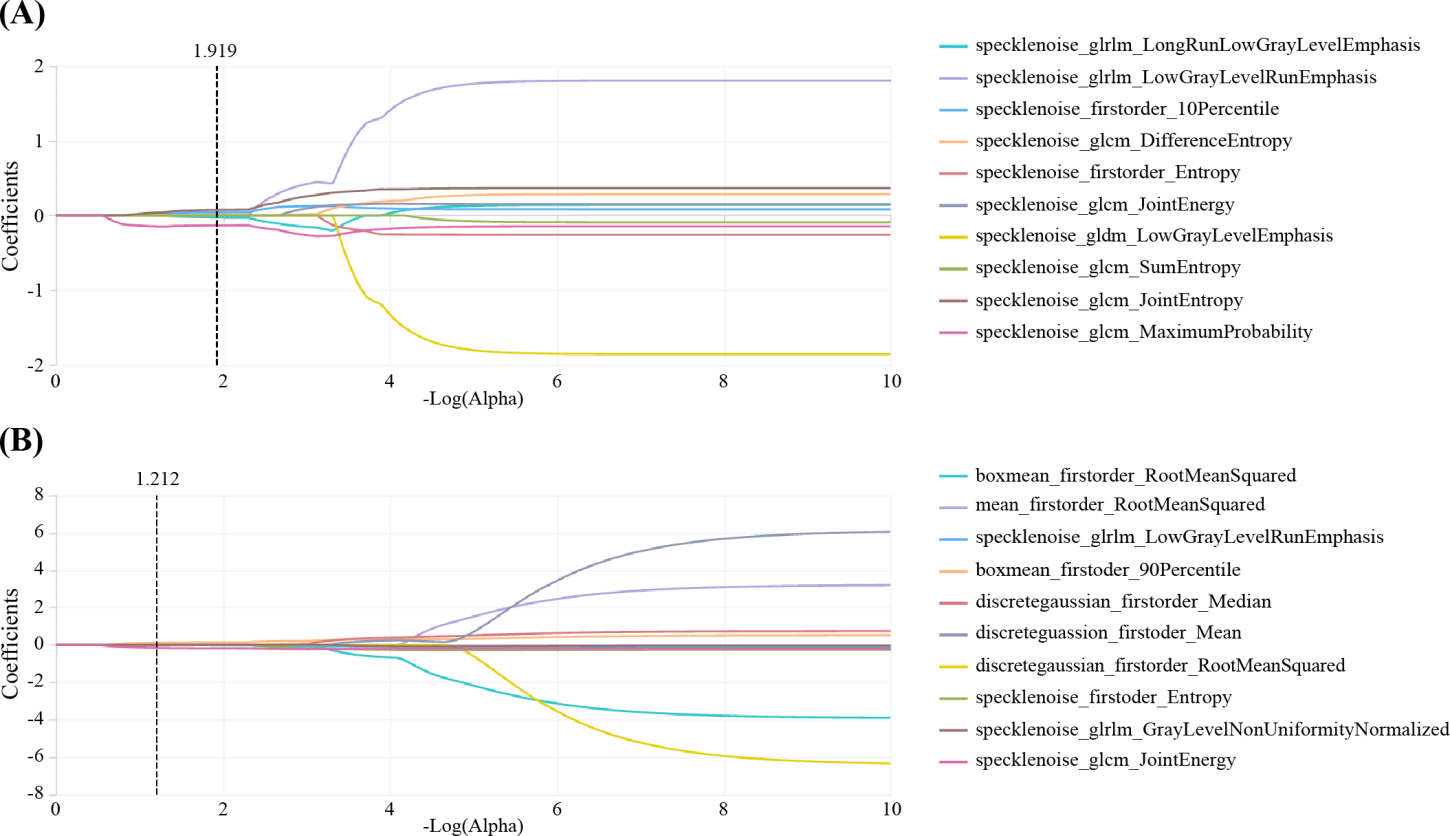
LASSO coefficient variation with hyperparameters for 10 features screened using the K-best method in AP 70kev images **(A)** and AP iodine-based MD images **(B)**. AP, arterial phase; LASSO, least absolute shrinkage and selection operator; VP, venous phase.

Among the models, the SVM model for the AP images demonstrated superior performance as the representative conventional radiomics model, achieving an AUC of 0.834 (95% CI, 0.692–0.977). The LASSO filtering process for the AP radiomics features is shown in **Figure 4A**.

### 3.4 DECT radiomics feature extraction and model development

In the Iodine-based image analysis, we extracted 540, 884, and 674 features with ICCs greater than 0.75 in the non-contrast, arterial, and venous phases, respectively. After screening, the top four features were selected for each phase, resulting in AUCs ranging from 0.767 to 0.907 for the training models and 0.638 to 0.703 for the test models. For the water-based image analysis, we extracted 647, 639, and 548 features with ICCs greater than 0.75 across the three phases. The final selection included two, three, and one key features, achieving AUCs ranging from 0.843 to 0.944 in the training models and from 0.823 to 0.925 in the test models.

**Figure 5:**
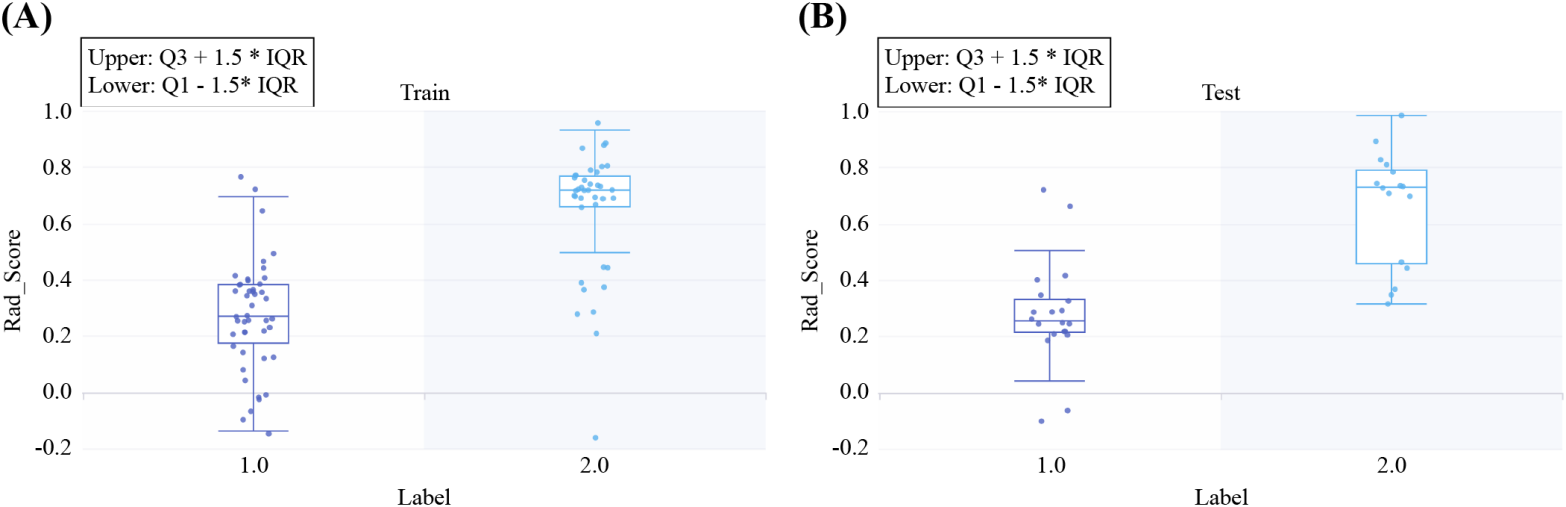
Rad-scores for all included samples in the training **(A)** and test **(B)** sets. Label 1 represents pleomorphic adenoma (PA), and Label 2 represents Warthin’s tumor (WT).

No significant differences were found among the LR, SVM, and RF models for any sequence. The LR model of the arterial-phase water-based images, which demonstrated the highest diagnostic accuracy in the test set, was selected as the final DECT radiomics model (**Supplementary Table S5**, **Supplementary Figures S5**-**S10**) with an AUC of 0.925 (95% CI, 0.844–0.999). The LASSO selection process for the arterial-phase water-based images is shown in **Figure 4B**, while **Figure 5** illustrates Rad-score differences among patients.

The Rad-score formula is as follows:


Rad−score=0.45+0.124×boxmean_firstorder_90Percentile−0.016× specklenoise_glrlm_LowGrayLevelRunEmphasis−0.149× specklenoise_glcm_JointEnergy

### 3.5 Nomogram development and assessment

**Figure 6:**
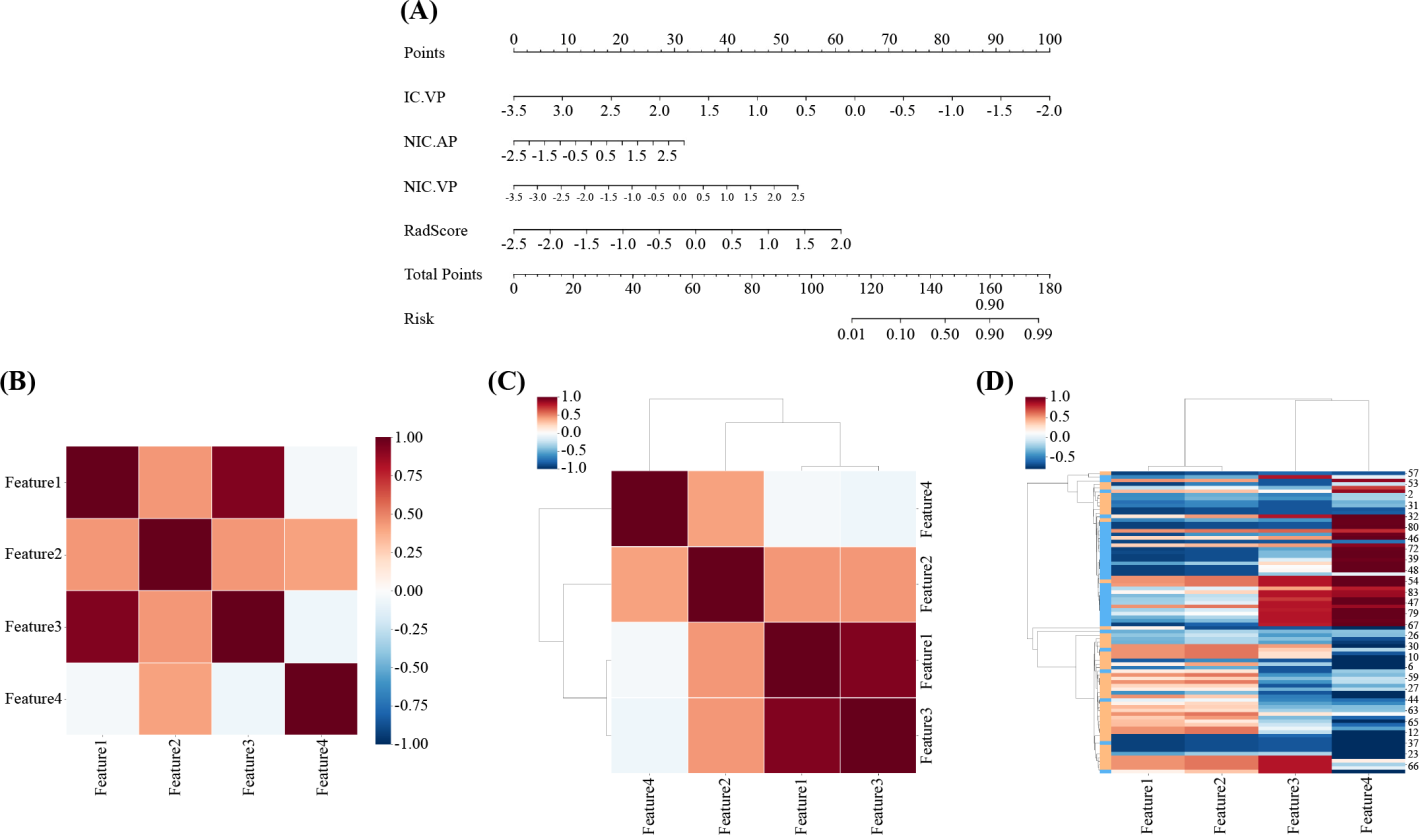
Radiomics nomogram was developed in the training set by combining IC(VP), NIC(AP), NIC(VP), and Rad-score. **(B, C)** Correlation heat maps for the combined model. **(D)** Unsupervised hierarchical clustering of the combined model’s features. Feature 1: IC(VP); Feature 2: NIC(AP); Feature 3: NIC(VP); Feature 4: Rad-score. AP, arterial phase; IC, iodine concentration; NIC, normalized iodine concentration; VP, venous phase.

**Figure 7:**
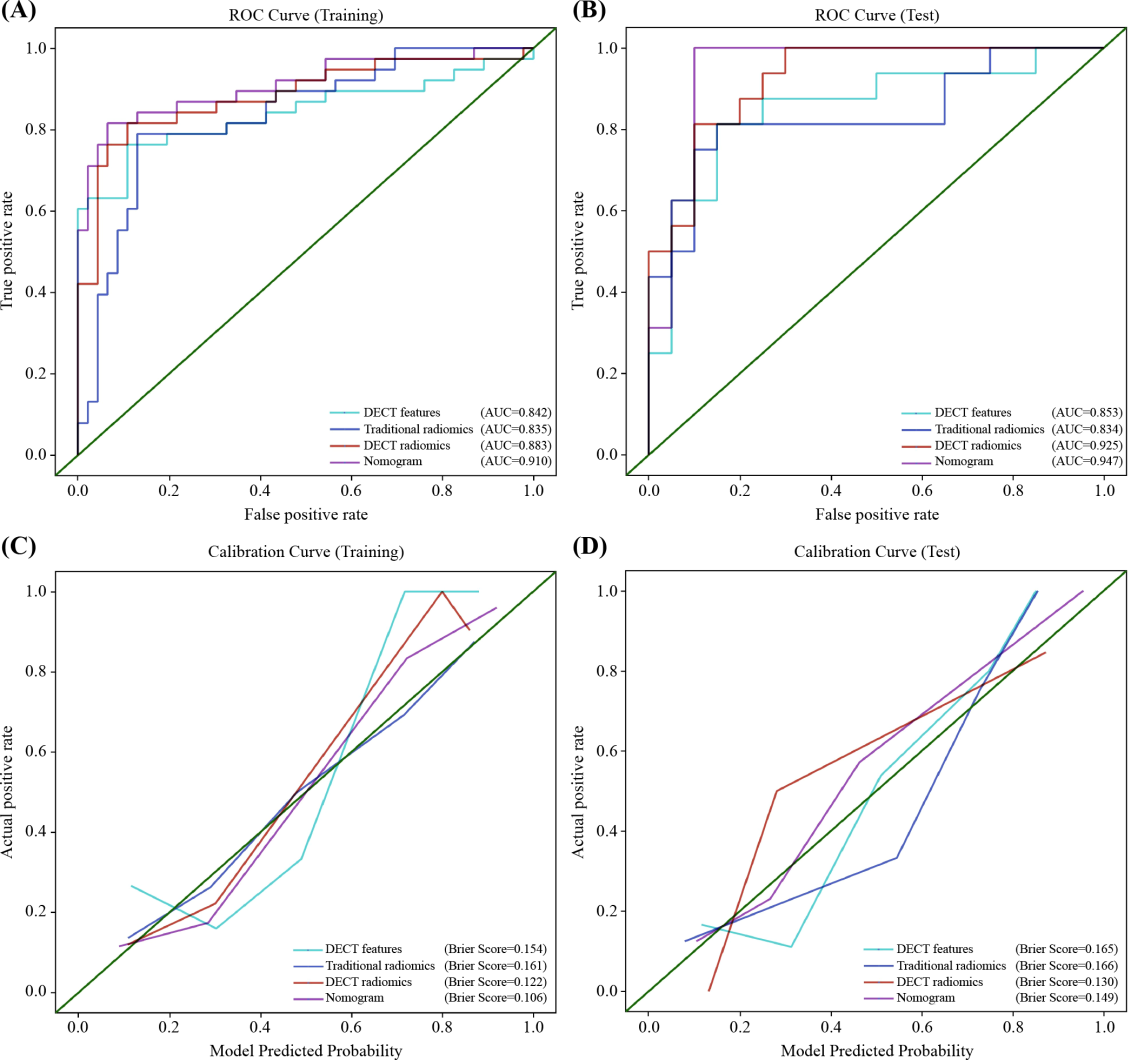
Receiver operator characteristics (ROC) and calibration curves for the DECT features model, traditional radiomics model, DECT radiomics model, and nomogram in the training **(A, C)** and test **(B, D)** sets. DECT, DECT, dual-energy CT.

**Figure 8:**
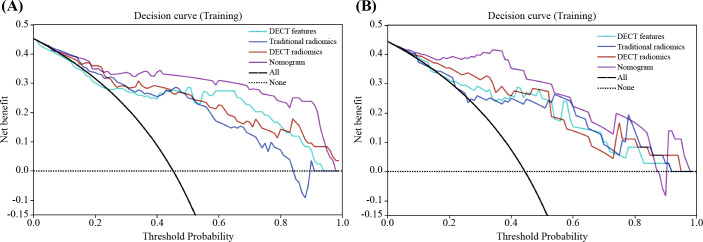
Decision curve analysis (DCA) for the classification of PA and WT in training **(A)** and test **(B)** sets. The nomogram provides the greatest net benefit compared to the other three datasets. PA, pleomorphic adenoma; PA, pleomorphic adenoma WT, Warthin’s tumor.

The nomogram for the combined model was constructed using IC (VP), NIC (AP), NIC (VP), and Rad scores derived from the arterial-phase water-based images (**Figure 6A**). The performance of each model is shown in **Supplementary Table S6** and **Supplementary Figure S11**. Heat maps indicate that the Rad score contributed the highest weight among the features (**Figures 6B–D**). A comparative analysis of the DECT features, traditional radiomics, and DECT radiomics is presented in **Table 4**, summarizing the combined model’s performance metrics. The ROC curves for all four models are depicted in **Figure 7**. The integrated model achieved AUCs of 0.91 in the training set and 0.947 in the test set, clearly outperforming both the DECT feature and traditional radiomics models. Although the AUC of the composite model was not significantly different from that of the DECT radiomics model, it exhibited greater specificity, accuracy, and precision. Calibration curves confirmed good alignment between predicted and actual outcomes for the training and test data (**Figure 7**). The DCA (**Figure 8**) demonstrates that the combined nomogram offers a higher net benefit across a wide range of threshold probabilities.

**Table 4:** Diagnostic performance of the four models in the training and test sets.

Model	AUC (95% CI)	Sensitivity (%)	Specificity (%)	Accuracy (%)	Precision (%)
Training set					
DECT features	0.842 (0.747–0.937)	0.763	0.891	0.833	0.853
Traditional radiomics	0.836 (0.748–0.924)	0.789	0.739	0.762	0.714
DECT radiomics	0.883 (0.805–0.961)	0.763	0.935	0.857	0.906
Nomogram	0.910 (0.842–0.977)	0.789	0.935	0.869	0.909
Test set					
DECT features	0.853 (0.718–0.988)	0.750	0.850	0.806	0.800
Traditional radiomics	0.834 (0.692–0.977)	0.812	0.750	0.778	0.722
DECT radiomics	0.925 (0.844–0.999)	0.688	0.900	0.806	0.846
Nomogram	0.947 (0.869–0.999)	0.812	0.900	0.861	0.867

DECT, dual-energy CT; AUC, area under the receiver operating characteristic curve; CI, confidence interval.

## 4 Discussion

Age, sex, smoking history, and cystic degeneration all showed significant differences between adenolymphomas and pleomorphic adenomas (P<0.001). These findings are in line with earlier research that found adenolymphomas are more common in middle-aged and older men and that nicotine from tobacco is a significant pathogenetic factor in their development. Because of their faster growth rate, adenolymphomas are more likely to experience cystic degeneration and necrosis (33). The imaging presentations of various parotid gland tumors often overlap, making accurate diagnosis using conventional CT and MRI challenging. This diagnostic process heavily relies on the clinician’s expertise, limiting reliability. The diagnostic sensitivity of salivary gland tumors for FNAC varies greatly, ranging from 71 to 93%, according to several earlier studies (34). Aspiration is an invasive test, and there is a risk of facial nerve damage, which is a drawback that is hard to totally prevent. Previous studies have shown promising results by combining DECT with radiomics for conditions such as pancreatic and lung tumors (28–30). However, to our knowledge, the application of MD techniques combined with radiomics for parotid tumor classification has not been explored. In our study, we developed an MD image-based radiomics model. We constructed a nomogram by integrating DECT quantitative parameters with MD radiomics features to distinguish PA from WT in the parotid gland. Our results indicate that MD radiomics provides superior diagnostic accuracy compared to DECT features and traditional radiomics alone. The combined model showed further improved diagnostic accuracy and clinical utility over the standalone DECT feature, traditional radiomics, and DECT radiomics models. These results suggest that the nomogram could serve as a valuable tool for the noninvasive preoperative evaluation of PA and WT. Because the two cancers are treated differently, using the nomograms to distinguish between them helps with treatment decision-making.

Additionally, our study underscored the utility of DECT quantitative parameters in distinguishing between PA and WT. Specifically, IC (VP), NIC (AP), and NIC (VP) were identified as independent predictors. The IC obtained from iodine-based DECT images reflects tumor blood perfusion. We found that the NIC (AP) of WT was higher than that of PA, whereas IC (VP) and NIC (VP) were significantly lower, consistent with previous studies (35, 36). This phenomenon may be related to the pathological structures of the two tumors. WT is a well-vascularized benign tumor with a dense microvascular network and leaky blood vessels, resulting in rapid contrast agent uptake during the arterial phase and quick washout during the venous phase (37). In contrast, PA contains epithelial and mesenchymal components with abundant intercellular mucin, leading to slower contrast uptake due to limited vascularity (18). The combined AUCs of the three independent predictors for the training and test sets were 0.842 and 0.853, respectively, demonstrating the feasibility of using DECT quantitative parameters to differentiate PA from WT.

The 70 keV VMI can simulate the attenuation characteristics of conventional 120 kVp scans in head and neck imaging, making them suitable alternatives for radiomic analysis in this region (38). In our study, radiomic analysis using 70 keV images demonstrated superior diagnostic efficacy during the arterial phase compared to other phases, with a sensitivity and specificity of 0.812 and 0.75, respectively, in the test set. Previous studies have highlighted the potential of traditional CT radiomics in distinguishing PA from WT (23, 39). Feng et al. identified the arterial phase as the most informative for differentiating these tumors, with their findings generally aligning with our results (22). Similarly, Jung et al. found no significant difference between quantitative enhancement analysis and radiomics in distinguishing PA from WT (40). This aligns with our results, where there was no significant difference between the two methods, although traditional CT radiomics performed slightly worse than the DECT feature model.

Radiomics enhances the analysis of MD images in DECT by quantifying the distribution of substances such as iodine and water into voxel relationships, offering a more precise and scientific. In our study, after analyzing radiomic features from both iodine- and water-based images across different phases, we found that the water-based images in the arterial phase yielded the highest diagnostic performance. The DECT radiomics model significantly improved the differential diagnosis of PA and WT compared to traditional CT radiomics. Water-based imaging, achieved through post-processing techniques that remove iodine signals, reflects the relative water distribution in tissues. We hypothesize that variations in the amount and distribution of water within parotid malignancies led to the maximum diagnostic efficacy of water-based images, which are based on the imaging principle of eliminating iodine and emphasizing water. The fact that Michela Gabelloni et al. were able to identify pleomorphic adenomas and adenolymphomas of the parotid gland using radiomics taken from T2WI sequences in MRI with similarly high sensitivity, specificity, and accuracy supports the significant difference in water content between the two benign tumors (41). This discrepancy might be explained by the fact that adenolymphomas, which have closely packed cells and many mesenchymal components, including tiny blood vessels, do not have a significant water content, while the majority of pleomorphic adenomas have a high and widely distributed water content due to their high mucus component content (42). This approach, often referred to as virtual non-enhanced imaging, closely mirrors the resolution and contrast of conventional non-enhanced image (43, 44). Notably, in the present study, the radiomic features derived from water-based images in the arterial phase outperformed those from the non-enhanced phase. We hypothesize that the reason for this phenomenon may be that virtual non-contrast images are derived from enhanced images that are consistent with both the position taken by the patient during scanning and the level of the tumor display, whereas real non-enhanced images are difficult to match exactly, leading to differences in final model performance. This also suggests that virtual non-enhanced images could serve as a viable substitute for conventional non-enhanced scans, thereby reducing redundant imaging and minimizing patient radiation exposure. Interestingly, the radiomic features from iodine-based images across all phases were less effective and even inferior to those of traditional CT radiomics. This may be attributed to the fact that iodine-based images primarily reflect iodine distribution, neglecting the contributions of other substances. As a result, the contrast between tissues and lesions is limited, leading to suboptimal image resolution and weaker diagnostic performance. And it also indicates that iodine might have interfered with the contrast-enhanced image histology model, making it perform marginally worse overall than the virtual non-enhanced images.

Studies have demonstrated that LASSO-based feature selection offers significant advantages in the differential diagnosis of parotid tumors (45). In our study, three key radiomic features—firstorder-90Percentile, glcm-Joint Energy, and glrlm-Low Gray Level Run Emphasis—were identified from arterial-phase water-based images as the most robust discriminators. These features were extracted from images processed using boxmean and speckle noise filters, which enhance edge definition and texture details. The boxmean filter implements fast rectangular homogenization, while the speckle noise filter introduces noise proportional to pixel intensity, thereby refining texture analysis. The first-order features capture voxel intensity distributions within a ROI, with the 90Percentile representing the voxel intensity at the 90th percentile. GLCM features, such as Joint Energy, describe the spatial relationships between voxel pairs, where higher energy values indicate greater uniformity in gray-level distribution. GLRLM, such as Low Gray Level Run Emphasis measures the prevalence of consecutive low-intensity pixels, reflecting textural uniformity and heterogeneity (46). These features can indicate tumor heterogeneity through the uniformity of the texture. Consistent with prior studies, WT exhibits higher texture heterogeneity owing to its complex cellular makeup, which includes epithelial components and lymphoid stroma, resembling the patterns often seen in malignant tumors (37, 47). Furthermore, the higher Low Gray Level Run Emphasis in WT than in PA suggests a greater presence of low-intensity voxels, likely due to the cystic and necrotic regions commonly found in WT. This observation aligns with our findings, which show that cystic lesions are more frequent in WT than in PA, as noted in the clinical characteristics of our patient cohort.

LR is one of the most commonly used machine learning algorithms, applying the principles of linear regression of continuous variables and using a logistic function to predict categorical outcomes. In contrast, RF algorithm, which relies on ensemble learning via decision trees, often underperforms when applied to small datasets owing to attribute perturbation, leading to large generalization errors (48, 49). SVMs are generalized linear classifiers that binarily classify data in a supervised learning manner and can use slack variables to avoid the influence of linearly indistinguishable data on the results; thus, a smaller set of samples achieves the greatest possible effectiveness (50, 51). In our study, although the differences in performance among the LR, SVM, and RF models were not significant, the LR model exhibited slightly higher AUC values across both the DECT features and radiomics models. This suggests that the classification problem at hand is primarily linear, explaining the marginally lower performance of the nonlinear RF and SVM models. To our knowledge, this is the first study comparing the effectiveness of multiple machine-learning models in distinguishing between PA and WT parotid tumors. A previous study by Yu et al. indicated that radiomic features from enhanced images often follow nonlinear patterns, favoring SVM and RF models over LR (45). However, Zhiying et al. found that the XGBoost algorithm outperformed SVM in classifying radiomics features derived from T1WI and T2WI MRI sequences (52).

Our study has several limitations. First, it is based on a single-center dataset with a small sample size, which restricts the robustness of the test results. Multi-center studies with larger datasets are needed to validate these findings. Second, we compared only three algorithms—LR, RF, and SVM—while other machine learning techniques, such as k-nearest neighbor (KNN) or XGBOOST, were not evaluated. Third, our focus was limited to MD technology in radiomics, excluding other DECT techniques, such as virtual monochromatic imaging, which warrant further investigation. Furthermore, there are few prior studies on radiomics in the parotid gland, the evidence is mixed, and there are variations in scanning parameters between clinical practice and data collection. As a result, it is challenging to create a consistent standard, and there are some restrictions when applying it in a clinical setting. In the future, we will increase the sample size for additional validation. Finally, we analyzed only two common benign parotid tumors, excluding other tumor types. Future studies should investigate multiple parotid tumors to provide a more comprehensive analysis.

## 5 Conclusion

DECT quantitative features are valuable in discriminating between the two most common parotid tumors. Additionally, water-based radiomics models derived from arterial phase images outperform traditional radiomics models. Our novel nomogram, which integrates DECT quantitative features with arterial phase water-based radiomics, further improves diagnostic accuracy. As a non-invasive preoperative tool, this model provides more precise guidance for individualized clinical treatment strategies.

## Data Availability

The raw data supporting the conclusions of this article will be made available by the authors, without undue reservation.
