# Supplementary material for: Nomogram combining dual-energy computed tomography features and radiomics for differentiating parotid warthin tumor from pleomorphic adenoma: a retrospective study
Source: Front Oncol. 2025 Mar 4;15:1505385. doi: 10.3389/fonc.2025.1505385 (PMC11914106; doi:10.3389/fonc.2025.1505385)
Supplement: Supplementary file 1 [file DataSheet1.docx]

Supplementary Material

# Supplementary Tables

Supplementary Table 1. Comparison of DECT features between the training and test sets

| DECT features | Training set  n = 84 | Test set  n = 36 | p-value |
| --- | --- | --- | --- |
| Attenuation (NP), HU ^a^ | 39.18 (30.71, 45.78) | 45.52 (31.65, 52.38) | 0.247 |
| Attenuation (AP), HU ^a^ | 63.25 (45.66, 84.19) | 57.58 (39.33, 84.79) | 0.392 |
| Attenuation (VP), HU ^a^ | 74.80 (66.23, 83.94) | 78.48 (60.67, 89.59) | 0.416 |
| Enhancement degree (AP), HU ^a^ | 21.49 (10.42, 41.68) | 12.12 (5.29, 34.44) | 0.069 |
| Enhancement degree (VP), HU ^a^ | 34.27 (27.04, 40.99) | 32.42 (28.94, 44.33) | 0.674 |
| IC (NP), 100 μg/cm³ ^b^ | -9.74 ± 2.64 | -9.48 ± 2.33 | 0.607 |
| IC (AP), 100 μg/cm³ ^a^ | -2.15 (-4.88, 4.88) | -4.03 (-7.02, 3.42) | 0.245 |
| IC (VP), 100 μg/cm³ ^a^ | 0.10 (-2.67, 4.71) | 0.27 (-2.86, 4.93) | 0.814 |
| NIC (AP), % ^a^ | -2.73 (-6.14, 7.36) | -4.35 (-8.71, 4.09) | 0.378 |
| NIC (VP), % ^a^ | 0.52 (-13.31, 19.29) | 1.36 (-14.37, 15.93) | 0.680 |

DECT, dual-energy CT; HU, Hounsfield unit; NP, non-enhanced phase; AP, arterial phase; VP, venous phase; IC, iodine concentration; NIC, normalized iodine concentration; a, Mann-Whitney U test; b, Student’s t-test.

Supplementary Table 2. Performance of the DECT feature model

| Feature | | Algorithm | AUC | |  | Sensitivity | |  | Specificity | |  | Accuracy | |  | Precision | |
| --- | --- | --- | --- | --- | --- | --- | --- | --- | --- | --- | --- | --- | --- | --- | --- | --- |
|  |  |  | Train | Test |  | Train | Test |  | Train | Test |  | Train | Test |  | Train | Test |
| DECT features | | LR | 0.842 | 0.853 |  | 0.763 | 0.750 |  | 0.891 | 0.850 |  | 0.833 | 0.806 |  | 0.853 | 0.800 |
|  |  | RF | 0.955 | 0.847 |  | 0.711 | 0.625 |  | 0.957 | 0.800 |  | 0.845 | 0.722 |  | 0.931 | 0.714 |
|  |  | SVM | 0.840 | 0.841 |  | 0.711 | 0.812 |  | 0.935 | 0.850 |  | 0.833 | 0.833 |  | 0.900 | 0.812 |

DECT, dual-energy CT; AUC, area under the receiver operating characteristic curve.

Supplementary Table 3. Selection results for radiomic features

| CT scan sequence | Number of features | | Radiomics feature | Coefficients |
| --- | --- | --- | --- | --- |
| Non-enhanced phase | | 2 | additivegaussiannoise_firstorder_90Percentile | 0.198 |
|  |  |  | laplaciansharpening_firstorder_90Percentile | 0.128 |
| Arterial phase | | 4 | specklenoise_firstorder_10Percentile | 0.040 |
|  |  |  | specklenoise_glcm_JointEntropy | 0.074 |
|  |  |  | specklenoise_glcm_MaximumProbability | -0.134 |
|  |  |  | specklenoise_glrlm_LongRunLowGrayLevelEmphasis | -0.028 |
| Venous phase | | 4 | log_firstorder_log-sigma-4-0-mm-3D-10Percentile | 0.053 |
|  |  |  | log_glcm_log-sigma-0-5-mm-3D-SumEntropy | 0.006 |
|  |  |  | wavelet_glcm_wavelet-LHL-ClusterShade | -0.067 |
|  |  |  | wavelet_glcm_wavelet-HLL-Idm | -0.067 |
| Iodine-based image (NP) | | 4 | log_firstorder_log-sigma-4-0-mm-3D-RootMeanSquared | -0.088 |
|  |  |  | log_glcm_log-sigma-2-0-mm-3D-ClusterShade | 0.103 |
|  |  |  | log_glcm_log-sigma-2-0-mm-3D-Imc1 | -0.024 |
|  |  |  | wavelet_glcm_wavelet-HLL-MaximumProbability | -0.005 |
| Iodine-based image (AP) | | 4 | normalize_gldm_DependenceNonUniformityNormalized | 0.150 |
|  |  |  | laplaciansharpening_glszm_HighGrayLevelZoneEmphasis | -0.407 |
|  |  |  | laplaciansharpening_glszm_SmallAreaHighGrayLevelEmphasis | 0.379 |
|  |  |  | laplaciansharpening_glszm_ZoneEntropy | 0.159 |
| Iodine-based image (VP) | | 4 | additivegaussiannoise_ngtdm_Contrast | -0.066 |
|  |  |  | log_firstorder_log-sigma-0-5-mm-3D-10Percentile | 0.052 |
|  |  |  | wavelet_gldm_wavelet-HLL-DependenceVariance | -0.102 |
|  |  |  | wavelet_gldm_wavelet-LLL-SmallDependenceEmphasis | -0.044 |
| Water-based image (NP) | | 2 | specklenoise_firstorder_Entropy | 0.084 |
|  |  |  | specklenoise_glcm_JointEnergy | -0.082 |
| Water-based image (AP) | | 3 | boxmean_firstorder_90Percentile | 0.124 |
|  |  |  | specklenoise_glcm_JointEnergy | -0.149 |
|  |  |  | specklenoise_glrlm_LowGrayLevelRunEmphasis | -0.016 |
| Water-based image (VP) | | 1 | specklenoise_firstorder_InterquartileRange | 0.038 |

NP, non-enhanced phase; AP, arterial phase; VP, venous phase.

Supplementary Table 4. Performance of all traditional radiomics models

| Sequence | Phase | Algorithm | AUC | |  | Sensitivity | |  | Specificity | |  | Accuracy | |  | Precision | |
| --- | --- | --- | --- | --- | --- | --- | --- | --- | --- | --- | --- | --- | --- | --- | --- | --- |
|  |  |  | Train | Test |  | Train | Test |  | Train | Test |  | Train | Test |  | Train | Test |
| 70 kev VMI | NP | LR | 0.949 | 0.728 |  | 0.868 | 0.562 |  | 0.913 | 0.750 |  | 0.893 | 0.667 |  | 0.892 | 0.643 |
|  |  | RF | 0.971 | 0.664 |  | 0.868 | 0.562 |  | 0.935 | 0.800 |  | 0.905 | 0.694 |  | 0.917 | 0.692 |
|  |  | SVM | 0.948 | 0.725 |  | 0.868 | 0.562 |  | 0.913 | 0.750 |  | 0.893 | 0.667 |  | 0.892 | 0.643 |
|  | AP | LR | 0.837 | 0.831 |  | 0.605 | 0.500 |  | 0.870 | 0.900 |  | 0.750 | 0.722 |  | 0.793 | 0.800 |
|  |  | RF | 0.875 | 0.816 |  | 0.789 | 0.750 |  | 0.870 | 0.900 |  | 0.833 | 0.833 |  | 0.833 | 0.857 |
|  |  | SVM | 0.836 | 0.834 |  | 0.789 | 0.812 |  | 0.739 | 0.750 |  | 0.762 | 0.778 |  | 0.714 | 0.722 |
|  | VP | LR | 0.743 | 0.669 |  | 0.711 | 0.750 |  | 0.609 | 0.450 |  | 0.655 | 0.583 |  | 0.600 | 0.522 |
|  |  | RF | 0.867 | 0.662 |  | 0.684 | 0.562 |  | 0.848 | 0.550 |  | 0.774 | 0.556 |  | 0.788 | 0.500 |
|  |  | SVM | 0.725 | 0.712 |  | 0.658 | 0.688 |  | 0.609 | 0.550 |  | 0.631 | 0.611 |  | 0.581 | 0.550 |

AUC, area under the receiver operating characteristic curve; NP, non-enhanced phase; AP, arterial phase; VP, venous phase.

Supplementary Table 5. Performance of all DECT radiomics models

| Sequence | Phase | Algorithm | AUC | |  | Sensitivity | |  | Specificity | |  | Accuracy | |  | Precision | |
| --- | --- | --- | --- | --- | --- | --- | --- | --- | --- | --- | --- | --- | --- | --- | --- | --- |
|  |  |  | Train | Test |  | Train | Test |  | Train | Test |  | Train | Test |  | Train | Test |
| Iodine-based image | NP | LR | 0.767 | 0.700 |  | 0.789 | 0.625 |  | 0.609 | 0.650 |  | 0.690 | 0.639 |  | 0.625 | 0.588 |
|  |  | RF | 0.903 | 0.675 |  | 0.763 | 0.500 |  | 0.891 | 0.700 |  | 0.833 | 0.611 |  | 0.853 | 0.571 |
|  |  | SVM | 0.775 | 0.703 |  | 0.763 | 0.625 |  | 0.630 | 0.650 |  | 0.690 | 0.639 |  | 0.630 | 0.588 |
|  | AP | LR | 0.866 | 0.697 |  | 0.737 | 0.562 |  | 0.870 | 0.800 |  | 0.810 | 0.694 |  | 0.824 | 0.692 |
|  |  | RF | 0.907 | 0.659 |  | 0.789 | 0.562 |  | 0.913 | 0.700 |  | 0.857 | 0.639 |  | 0.882 | 0.600 |
|  |  | SVM | 0.870 | 0.666 |  | 0.763 | 0.562 |  | 0.870 | 0.650 |  | 0.821 | 0.611 |  | 0.829 | 0.562 |
|  | VP | LR | 0.826 | 0.638 |  | 0.763 | 0.562 |  | 0.739 | 0.650 |  | 0.750 | 0.611 |  | 0.707 | 0.562 |
|  |  | RF | 0.878 | 0.681 |  | 0.895 | 0.500 |  | 0.783 | 0.650 |  | 0.833 | 0.583 |  | 0.773 | 0.533 |
|  |  | SVM | 0.820 | 0.659 |  | 0.763 | 0.562 |  | 0.674 | 0.600 |  | 0.714 | 0.583 |  | 0.659 | 0.529 |
| Water-based image | NP | LR | 0.894 | 0.844 |  | 0.711 | 0.625 |  | 0.957 | 0.999 |  | 0.845 | 0.833 |  | 0.931 | 0.999 |
|  |  | RF | 0.904 | 0.848 |  | 0.737 | 0.688 |  | 0.935 | 0.950 |  | 0.845 | 0.833 |  | 0.903 | 0.917 |
|  |  | SVM | 0.897 | 0.837 |  | 0.658 | 0.500 |  | 0.957 | 0.999 |  | 0.821 | 0.778 |  | 0.926 | 0.999 |
|  | AP | LR | 0.883 | 0.925 |  | 0.763 | 0.688 |  | 0.935 | 0.900 |  | 0.857 | 0.806 |  | 0.906 | 0.846 |
|  |  | RF | 0.944 | 0.884 |  | 0.763 | 0.750 |  | 0.935 | 0.900 |  | 0.857 | 0.833 |  | 0.906 | 0.857 |
|  |  | SVM | 0.880 | 0.888 |  | 0.763 | 0.688 |  | 0.935 | 0.900 |  | 0.857 | 0.806 |  | 0.906 | 0.846 |
|  | VP | LR | 0.843 | 0.884 |  | 0.737 | 0.750 |  | 0.848 | 0.900 |  | 0.798 | 0.833 |  | 0.800 | 0.857 |
|  |  | RF | 0.891 | 0.823 |  | 0.711 | 0.500 |  | 0.891 | 0.900 |  | 0.810 | 0.722 |  | 0.844 | 0.800 |
|  |  | SVM | 0.843 | 0.884 |  | 0.737 | 0.750 |  | 0.848 | 0.900 |  | 0.798 | 0.833 |  | 0.800 | 0.857 |

DECT, dual-energy CT; AUC, area under the receiver operating characteristic curve; NP, non-enhanced phase; AP, arterial phase; VP, venous phase.

Supplementary Table 6. Performance of the nomogram

| Feature | | Algorithm | AUC | |  | Sensitivity | |  | Specificity | |  | Accuracy | |  | Precision | |
| --- | --- | --- | --- | --- | --- | --- | --- | --- | --- | --- | --- | --- | --- | --- | --- | --- |
|  |  |  | Train | Test |  | Train | Test |  | Train | Test |  | Train | Test |  | Train | Test |
| Combined features | | LR | 0.910 | 0.947 |  | 0.789 | 0.812 |  | 0.935 | 0.900 |  | 0.869 | 0.861 |  | 0.909 | 0.867 |
|  |  | RF | 0.960 | 0.912 |  | 0.816 | 0.688 |  | 0.957 | 0.900 |  | 0.893 | 0.806 |  | 0.939 | 0.846 |
|  |  | SVM | 0.915 | 0.947 |  | 0.789 | 0.812 |  | 0.935 | 0.900 |  | 0.869 | 0.861 |  | 0.909 | 0.867 |

AUC, area under the receiver operating characteristic curve; LR, logistic regression; RF, random forest; SVM, support vector machine.

# Supplementary Figures

.

| **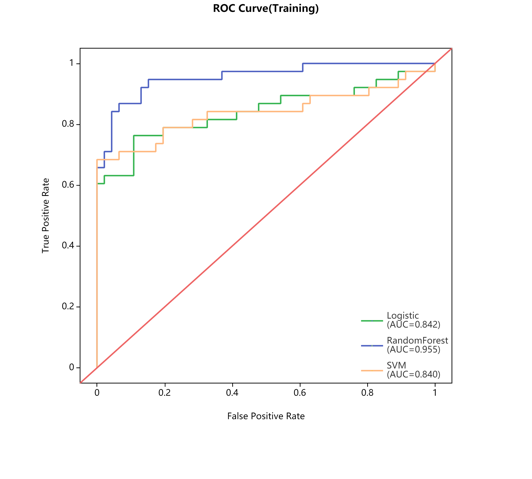** | **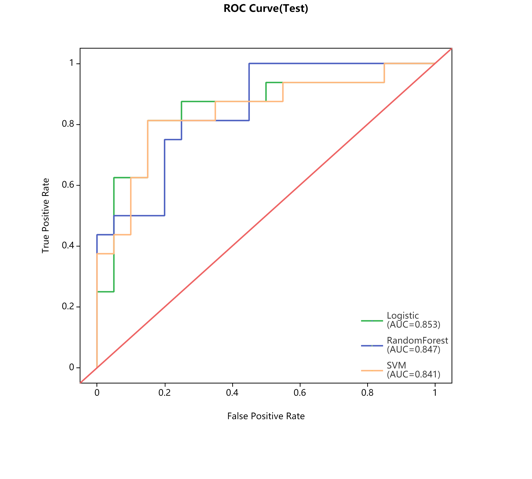** |
| --- | --- |
| **A** | **B** |

**Supplementary Figure 1.** ROC curves of the LR, RF, and SVM models of DECT features in the training (A) and test (B) datasets. ROC, receiver operating characteristic; DECT, dual-energy CT; AUC, area under the receiver operating characteristic curve.

| **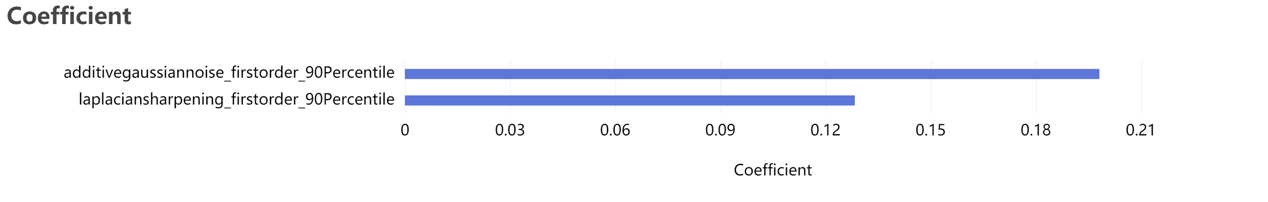** | |
| --- | --- |
| **A** | |
| **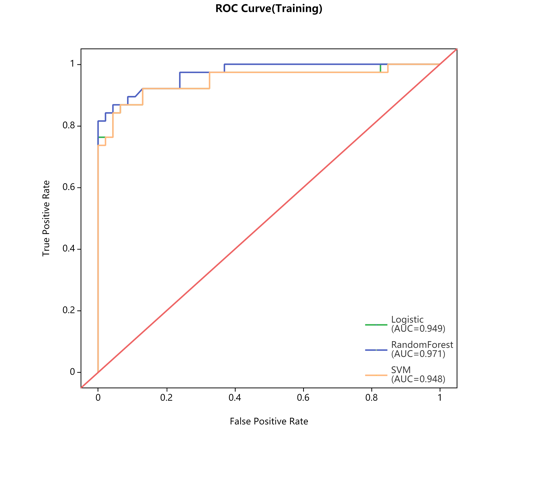** | **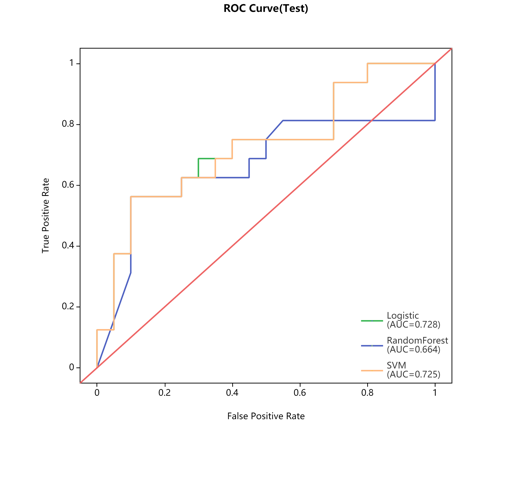** |
| **B** | **C** |

**Supplementary Figure 2.** Features selected for 70 kev VMI in the non-enhanced phase (A) and ROC curves of LR, RF, and SVM models in the training (B) and test datasets (C). VMI: virtual monochromatic image. ROC, receiver operating characteristic; AUC, area under the receiver operating characteristic curve.

| **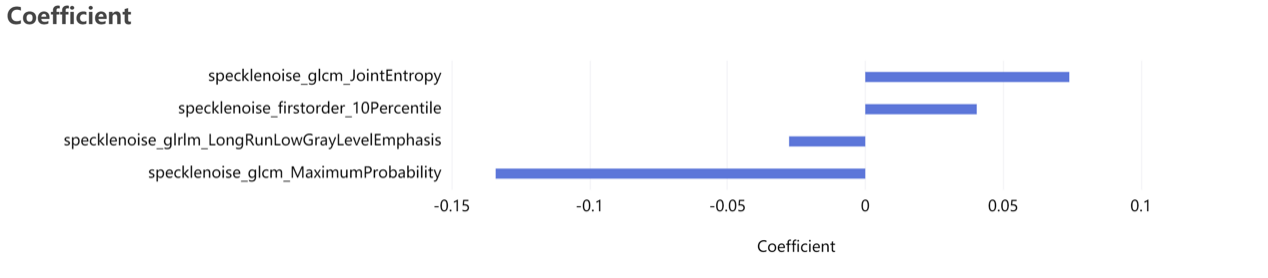** | |
| --- | --- |
| **A** | |
| **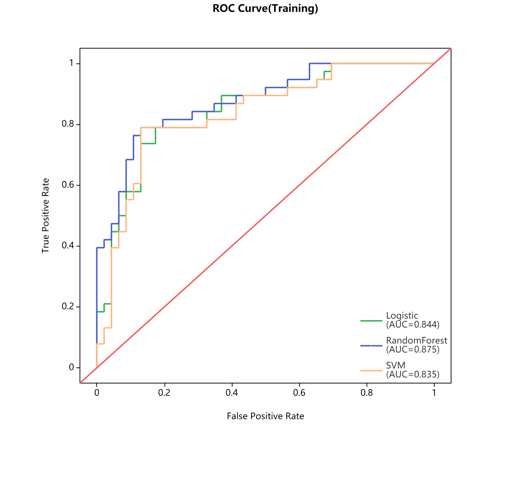** | **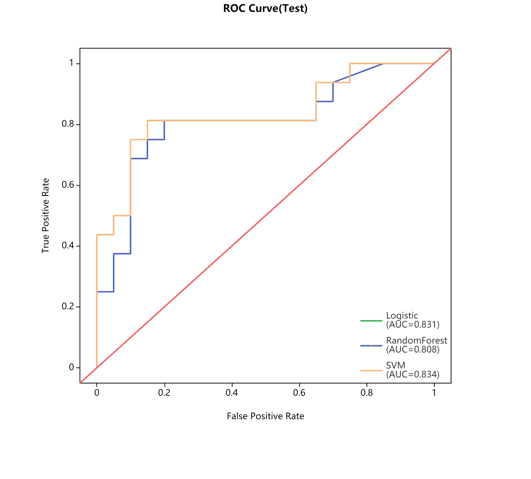** |
| **B** | **C** |

**Supplementary Figure 3.** Features selected for 70 kev VMI in the arterial phase (A) and ROC curves of LR, RF, and SVM models in the training (B) and test datasets (C). ROC, receiver operating characteristic; AUC, area under the receiver operating characteristic curve.

| **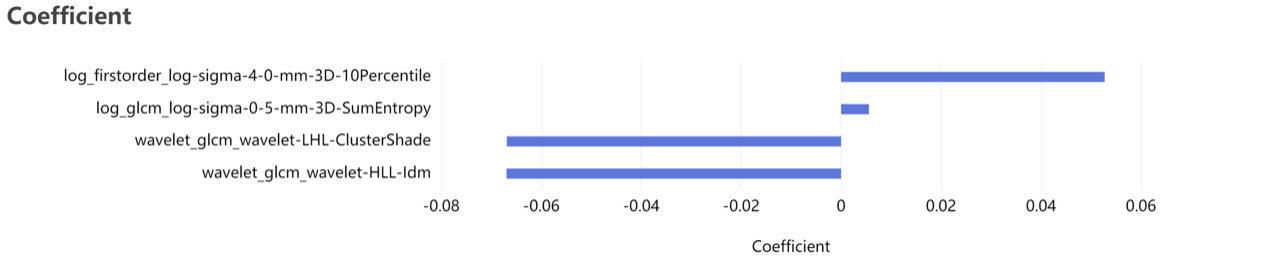** | |
| --- | --- |
| **A** | |
| **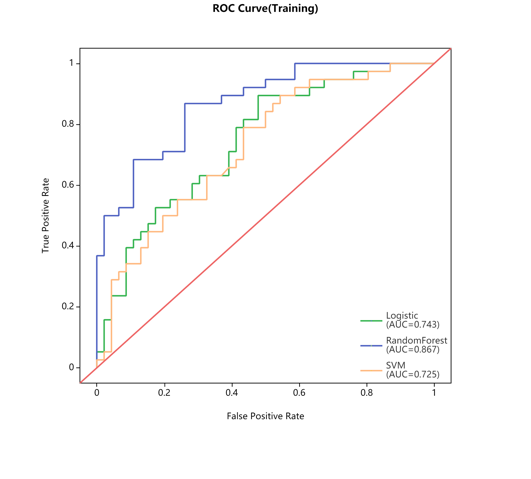** | **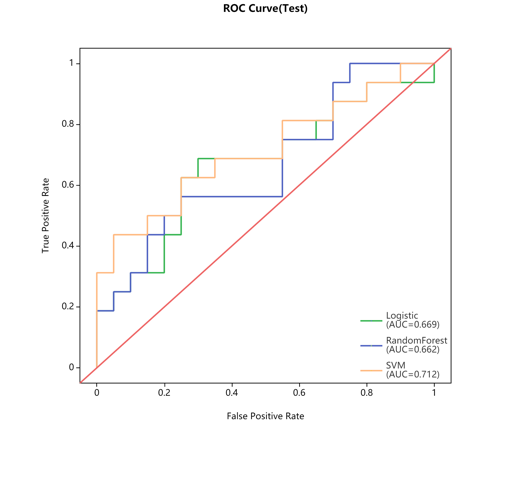** |
| **B** | **C** |

**Supplementary Figure 4.** Features selected for 70 kev VMI in the venous phase (A) and ROC curves of LR, RF, and SVM models in the training (B) and test datasets (C). ROC, receiver operating characteristic; AUC, area under the receiver operating characteristic curve.

| **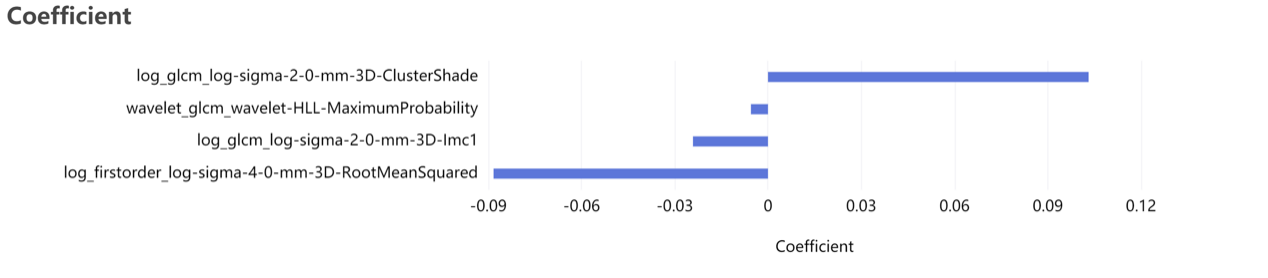** | |
| --- | --- |
| **A** | |
| **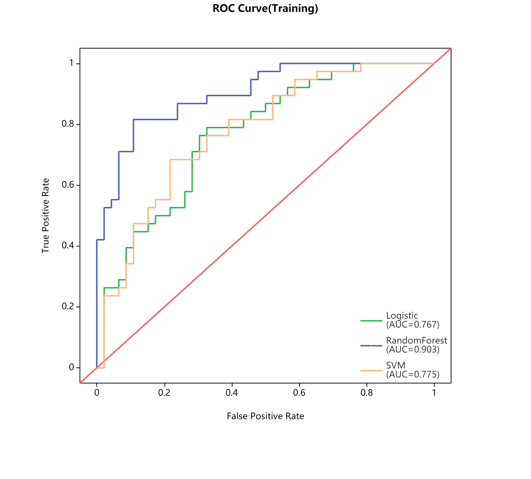** | **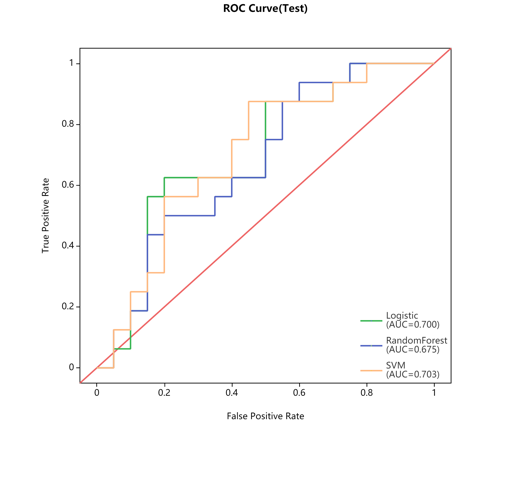** |
| **B** | **C** |

**Supplementary Figure 5.** Features selected for iodine-based images in the non-enhanced phase (A) and ROC curves of the LR, RF, and SVM models in the training (B) and test datasets (C). ROC, receiver operating characteristic; AUC, area under the receiver operating characteristic curve.

| **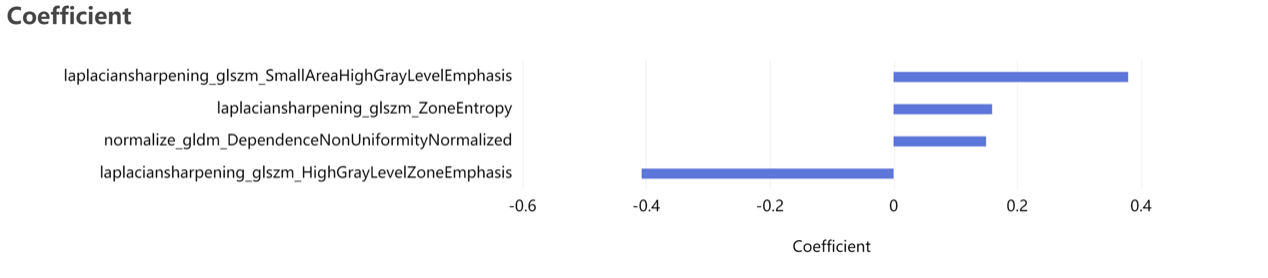** | |
| --- | --- |
| **A** | |
| **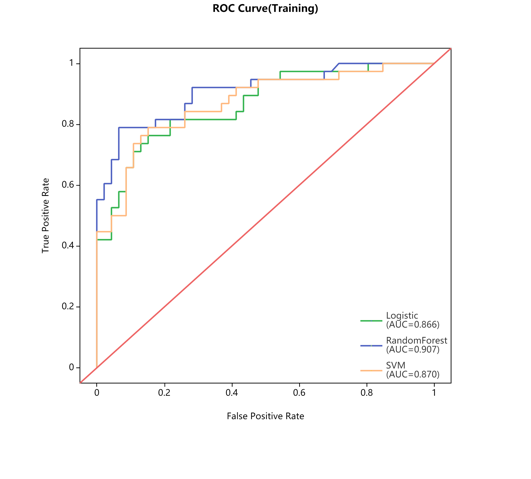** | **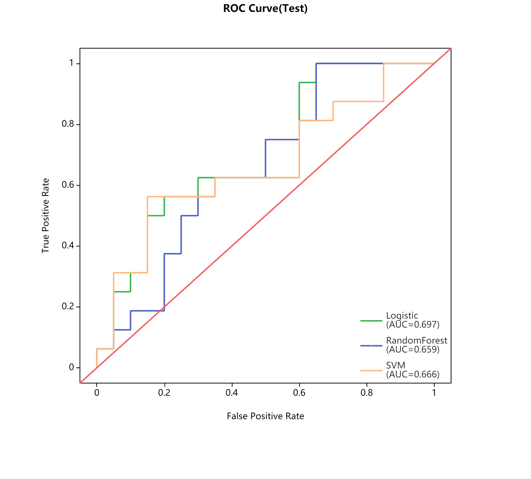** |
| **B** | **C** |

**Supplementary Figure 6.** Features selected for the iodine-based image in the arterial phase (A) and ROC curves of the LR, RF, and SVM models in the training (B) and test datasets (C). ROC, receiver operating characteristic; AUC, area under the receiver operating characteristic curve.

| **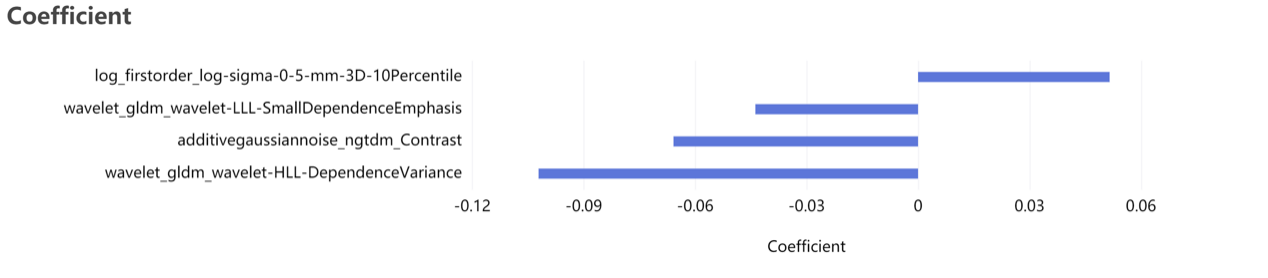** | |
| --- | --- |
| **A** | |
| **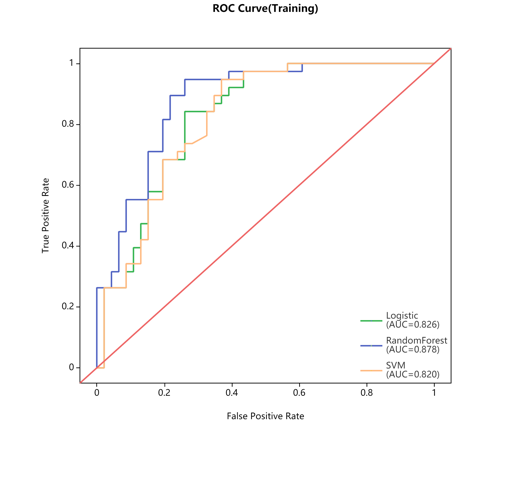** | **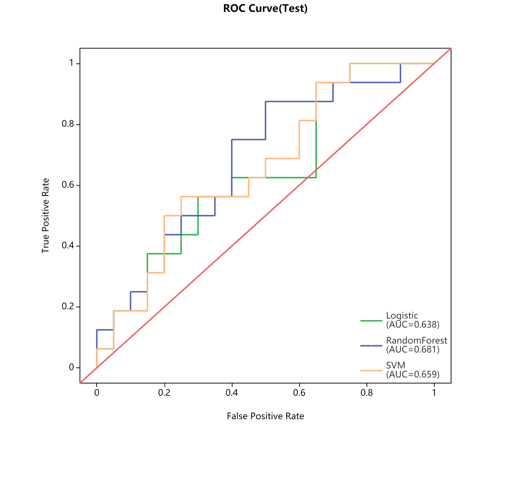** |
| **B** | **C** |

**Supplementary Figure 7.** Features selected for the iodine-based image in the venous phase (A) and ROC curves of the LR, RF, and SVM models in the training (B) and test datasets (C). ROC, receiver operating characteristic; AUC, area under the receiver operating characteristic curve.

| **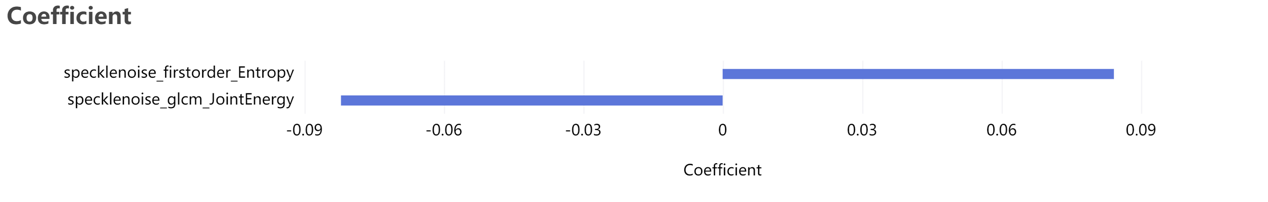** | |
| --- | --- |
| **A** | |
| **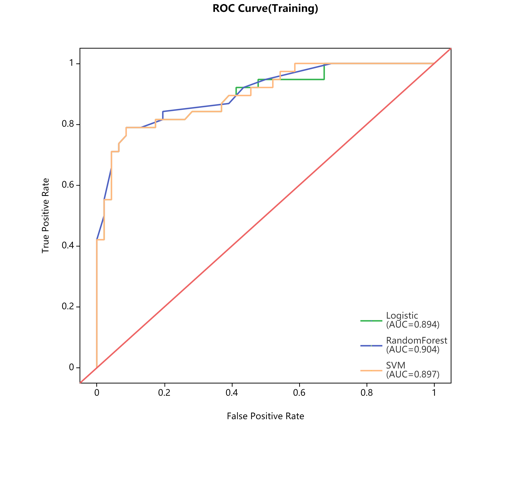** | **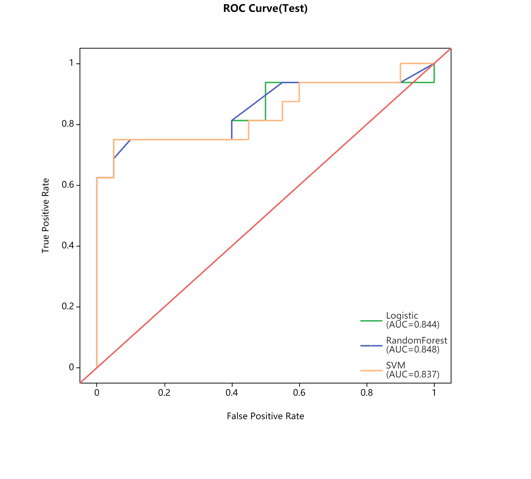** |
| **B** | **C** |

**Supplementary Figure 8.** Features selected for the water-based image in the nonenhanced phase (A) and ROC curves of the LR, RF, and SVM models in the training (B) and test datasets (C). ROC, receiver operating characteristic; AUC, area under the receiver operating characteristic curve.

| **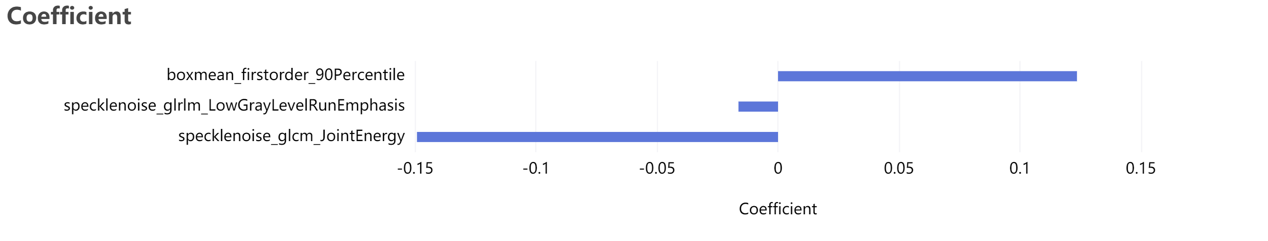** | |
| --- | --- |
| **A** | |
| **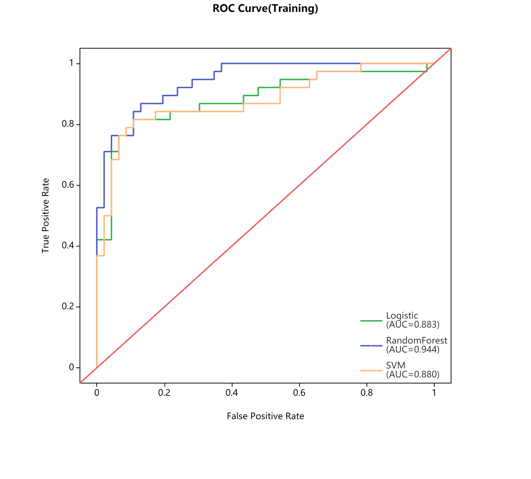** | **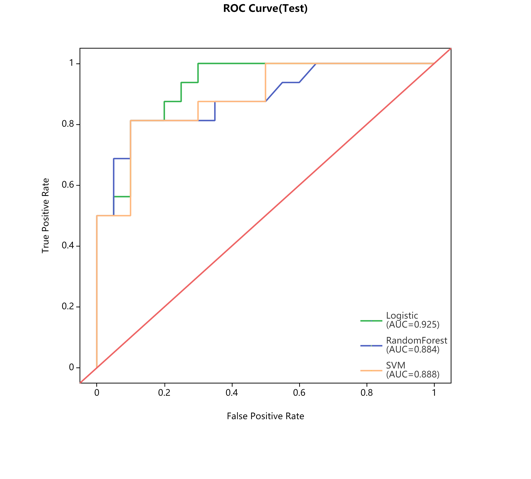** |
| **B** | **C** |

**Supplementary Figure 9.** Features selected for the water-based image in the arterial phase (A) and ROC curves of the LR, RF, and SVM models in the training (B) and test datasets (C). ROC, receiver operating characteristic; AUC, area under the receiver operating characteristic curve.

| **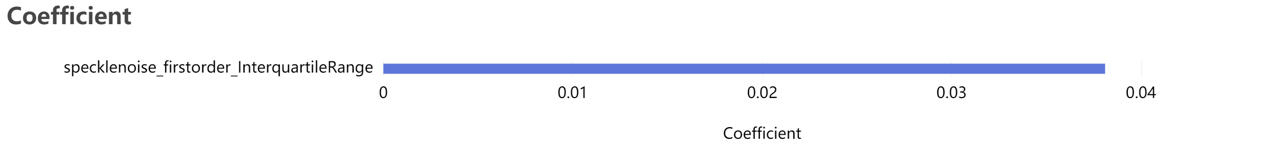** | |
| --- | --- |
| **A** | |
| **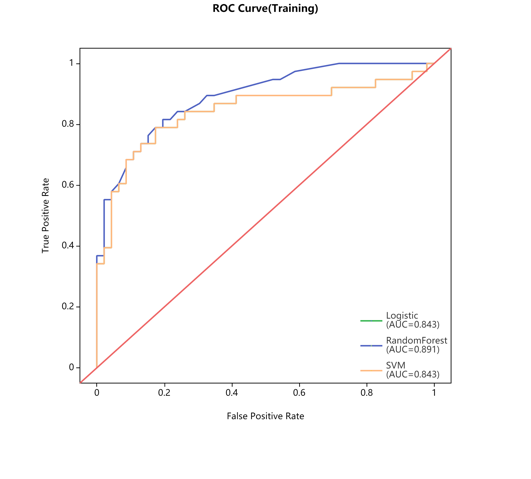** | **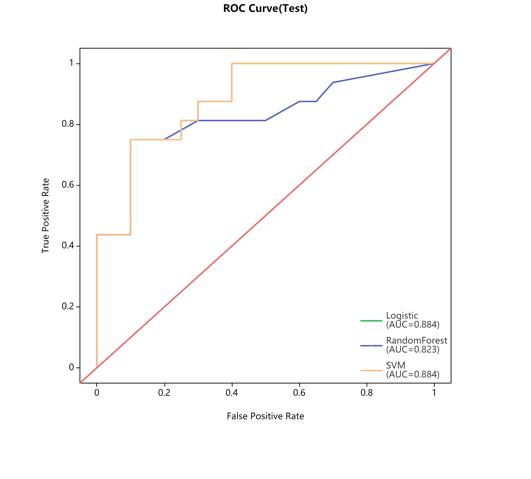** |
| **B** | **C** |

**Supplementary Figure 10.** Features selected for the water-based image in the venous phase (A) and ROC curves of the LR, RF, and SVM models in the training (B) and test datasets (C). ROC, receiver operating characteristic; AUC, area under the receiver operating characteristic curve.

| **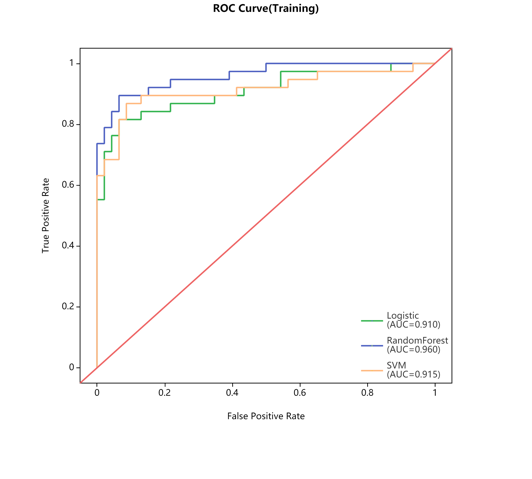** | **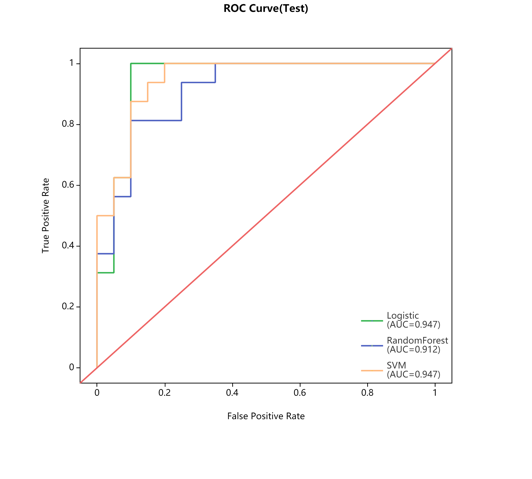** |
| --- | --- |
| **A** | **B** |

**Supplementary Figure 11.** ROC curves of the LR, RF, and SVM models of the nomogram. ROC, receiver operating characteristic; AUC, area under the receiver operating characteristic curve.
